# Application of radiotherapy-preexcited gambogic acid dual targeting nanoparticles in colorectal cancer

**DOI:** 10.1039/d5ra02815a

**Published:** 2025-12-03

**Authors:** Liuqi Sang, Qun Zhang, Zixin Liang, Xinyi Xu, Xueying Bai, Ning Wang, Xingzhi Han, Li Li, Jing Hu, Xiaoping Qian

**Affiliations:** a The Comprehensive Cancer Center, Nanjing Drum Tower Hospital Clinical College of Xuzhou Medical University Nanjing 210008 China; b Department of Oncology, Nanjing Drum Tower Hospital Clinical College of Traditional Chinese and Western Medicine, Nanjing University of Chinese Medicine 321 Zhongshan Road Nanjing Jiangsu 210008 China xiaopingqian@nju.edu.cn doctorhujing@163.com +86-25-68182342 +86-13951743162 +86-13057668736; c Comprehensive Cancer Centre of Drum Tower Hospital, Medical School of Nanjing University, Clinical Cancer Institute of Nanjing University Nanjing 210008 China

## Abstract

Delivering anti-tumor drugs to the correct location is an important strategy for improving tumor treatment efficacy and reducing side effects. To this end, this study developed a P-selectin-targeted drug delivery platform, where fucoidan-modified PLGA nanoparticles were loaded with the anti-tumor drug gambogic acid (GA) to form a novel nanoparticle, Fucoidan-PLGA/GA NPs (FPG). Fucoidan serves as a natural hydrophilic shell, enabling specific dual-targeting by binding to P-selectin overexpressed on tumor vascular endothelial cells and tumor cells, allowing FPG to cross the vascular barrier and reach the tumor tissue. *In vitro* and *in vivo* experimental results demonstrate that FPG has excellent binding capability to tumor cells and endothelial cells. The radiation-induced differential expression of P-selectin further enhances drug-targeted delivery. FPG combined with radiotherapy exhibited significant advantages in inhibiting tumor growth, inducing apoptosis, reducing tumor volume, modulating the immune microenvironment, and promoting cell phagocytosis. This targeted delivery system holds great potential for improving tumor treatment efficacy and reducing side effects in future cancer therapies.

## Abstract 2

Radiotherapy-induced expression of P-selectin, which subsequently promotes the targeted delivery of FPG.

## Graphical abstract

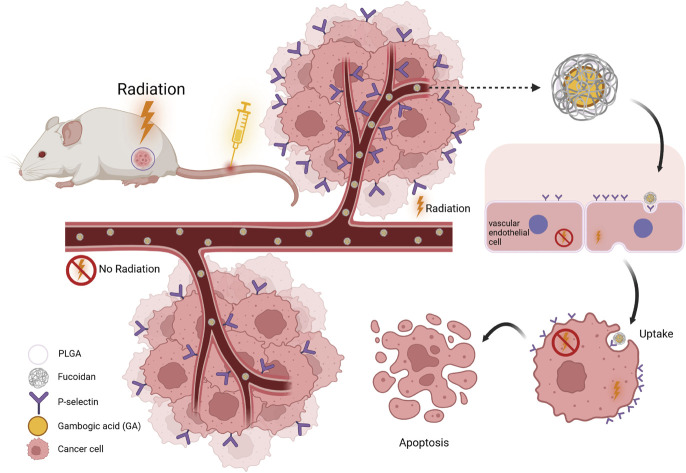

## Introduction

Colorectal cancer (CRC) remains the third leading cause of cancer-related deaths globally, with over 1.9 million new cases reported annually.^1^ In recent years, significant progress has been made in the early screening and treatment of colorectal cancer, particularly with the combined use of surgical resection, chemotherapy drugs, radiotherapy, and targeted therapy, which has effectively improved the overall survival rate of patients.^2^ However, the 5-year survival rate for advanced CRC remains stagnated at less than 15%.^3^ The dose-limiting toxicity of chemotherapy drugs and the high off-target rate of EPR-based nano-drugs for passive tumor targeting limit the efficacy against malignant tumors.^4,5^ Although traditional chemotherapy drugs have clear therapeutic effects in tumor treatment, their non-selective cytotoxicity often leads to severe adverse reactions (such as bone marrow suppression and organ damage).^6,7^ In contrast, natural anti-tumor drugs (such as plant-derived compounds) offer new options for optimizing the therapeutic window due to their unique regulatory properties and relatively low systemic toxicity. Among these, gambogic acid, a natural active compound extracted from gamboge resin, exhibits strong cytotoxicity against various tumors, including colorectal cancer.^8–10^

The structural uniqueness and poor water solubility of gambogic acid lead to limitations in its pharmacokinetic properties,^11^ The application of nanotechnology theoretically addresses these issues. Although the application of nanoparticle-based drugs has shown clinical advantages in reducing the life-threatening toxicity of chemotherapy and improving overall patient survival,^12^ the actual therapeutic efficacy of nanodrugs is much lower than expected, as their delivery rate into tumor cells is low when they encounter continuous biological barriers in the bloodstream, undergo transvascular migration, diffuse in tumor tissue, and are internalized by tumor cells. It is estimated that only approximately 0.7% of intravenously injected nanoparticles (NPs) reach tumor tissue, and only 2% of tumor cells are able to easily internalize NPs.^13,14^ One important reason for this is that drugs reaching the tumor site must first overcome the vascular system and cross the blood vessel barrier before they can directly interact with tumor cells. Nanodrugs in the bloodstream encounter the endothelial lining, which is the first physiological barrier and a critical biological barrier in cancer drug delivery.^15^ Single-target therapy approaches often fail to achieve the desired results. Therefore, the development of new, precise drug delivery systems capable of crossing the tumor vascular barrier and specifically targeting tumor cells has become a key research direction in the treatment of colorectal cancer.

The induced or overexpressed expression of new antigens and other biomarkers on the surface of cancer cells under external stimuli can help differentiate cancer cells from normal tissue.^16,17^ This can be followed by enhancing drug delivery efficiency through ligand–receptor binding, making this “planned delivery” approach a promising therapeutic strategy. P-selectin, as an adhesion molecule, is primarily expressed on activated platelets and endothelial cells. In recent years, researchers have observed a significant upregulation of P-selectin expression in various tumor tissues, especially in solid tumors, while its expression is low or absent in adjacent normal tissues. Additionally, this molecule increases further following radiation in the tumor microenvironment.^18,19^ This differential expression between tumor cells and endothelial cells makes P-selectin a promising target with broad application prospects. Fucoidan is a type of sulfated polysaccharide, primarily composed of fucose, and it can be extracted from various species of brown algae. It also maintains nanomolar affinity for P-selectin.^20,21^ Due to its excellent biocompatibility and unique biological activity, it has been widely used in the food and healthcare product industries.^22^

Unlike traditional single-target strategies, we have designed a colorectal cancer treatment model that uses radiotherapy as a preconditioning method. This model utilizes Fucoidan-modified PLGA nanoparticles loaded with the anti-tumor drug gambogic acid as the delivery medium. Radiotherapy is used to mark specific tumor regions, promoting the overexpression of target molecules, thus enabling precise treatment. This strategy achieves “dual targeting of both the endothelium and tumor” through the synergistic effect of radiotherapy-induced dynamic expression of target molecules and the specific binding of Fucoidan-P-selectin. It breaks through the vascular barrier while enhancing the uptake efficiency by tumor cells, thus reducing side effects and amplifying therapeutic efficacy.

## Materials and methods

### Materials

GA (purity ≥99%) and Fucoidan (≥98%, from brown seaweed) were purchased from Aladdin Industrial Corporation (Shanghai, China). PLGA (molecular weight 38 000–54,000) was purchased from Macklin (Shanghai, China). Roswell Park Memorial Institute (RPMI) 1640 medium, trypsin, Fetal bovine serum (FBS) were acquired from Gibco. Penicillin–streptomycin solution was purchased from Beyotime Biotechnology Company (Shanghai, China). Annexin V-FITC/PI apoptosis kit was purchased from Vazyme Biotech Co., Ltd (Nanjing, China). A reactive oxygen species assay kit (including 2,7-dichlorofluorescein diacetate (DCFH-DA)) was purchased from Biyuntian Biotechnology (Shanghai, China). Mouse antibodies, including CD3, CD8a, programmed death-1 antibodies (PD-1), CD11c, CD80, CD86, CD11b, and F4/80, all purchased from Biolegend (USA).

### Preparation of Fucoidan-PLGA/GA NPs

Fucoidan-PLGA/GA NPs were prepared using an improved emulsification and solvent evaporation method. An organic solvent containing PLGA (6 mg) and GA (2 mg) in 500 µL of dichloromethane was prepared and added to a 4 mL aqueous solution of fucoidan (0.5% w/v). An improved emulsification was achieved using an ultrasonic processor (Sonics & Materials Inc., USA, Model VCX 130, S/N61766T-02-11). The mixture was sonicated using a 6 mm diameter titanium probe operating at a frequency of 20 kHz. The process was conducted in pulse mode with a cycle of 3 seconds on and 3 seconds off for a total time of 6 minutes. The power amplitude was set to 45% of the device's maximum capacity (130 W), delivering an approximate output power of 58.5 W. To dissipate heat and prevent the degradation of components, the sample vessel was consistently maintained in an ice-water bath during the entire sonication procedure. Then the mixture forms a water-in-oil emulsion. The emulsion was stirred at room temperature for more than 2 hours in a ventilated area to remove the organic solvent, followed by washing the Fucoidan-PLGA/GA NPs three times with deionized water.

### Characterization of Fucoidan-PLGA/GA NPs

The encapsulation efficiency (EE) and drug loading (DL) of GA in Fucoidan-PLGA/GA nanoparticles were calculated using a UV-Vis spectrophotometer. The particle size, zeta potential, and polydispersity index (PDI) of Fucoidan-PLGA/GA nanoparticles were measured using a DLS analyzer (Zetasizer, Malvern, UK). The stability of the nanoparticles was evaluated by measuring their size and PDI over 72 hours. The morphology of Fucoidan-PLGA/GA nanoparticles was observed using transmission electron microscopy (TEM). For drug release studies, 1 mL of free gambogic acid and Fucoidan-PLGA/GA nanoparticles were dialyzed in 3 mL of PBS (pH 7.4, containing 0.5% wt of Tween 80) using a dialysis bag with a molecular weight cutoff of 10 kDa at 37 °C for 1 week, and the GA content was measured using high-performance liquid chromatography (HPLC) (Shimadzu, Japan). The chemical interaction between Fucoidan and PLGA was evaluated by FT-IR spectroscopy (Thermo Fisher Scientific, USA) and UV-vis spectroscopy (BioTeK, USA).

### *In vitro* cytotoxicity assay

CT26 and NCM460 cells were pre-seeded in a 96-well plate. For the combined radiotherapy group, CT26 cells were exposed to 4 Gy radiation after attachment, followed by overnight incubation. Then two different concentrations of gambogic acid nanoparticles were added, and cell viability was assessed using the CCK-8 assay kit after 24 hours to evaluate the cytotoxicity of FPG and PG.

### Apoptosis assay

CT26 cells were pre-seeded in a 6-well plate, and for the combined radiotherapy group, CT26 cells were exposed to 4 Gy radiation after cell attachment. After overnight incubation, the cells were cultured in medium containing two different nanoparticles (PG and FPG), both of which contained 0.4 µg mL^−1^ of gambogic acid (GA). After 24 hours, the cells were collected, treated with an apoptosis detection kit, and assessed and analyzed using a flow cytometer (CytoFLEX, Beckman, USA).

### ROS measurement

CT26 cells were seeded into confocal dishes and cultured overnight. For the combination therapy group, cells were subjected to a 2 Gy dose of radiation after adhesion. Following the respective treatments for each group, cells were incubated with nanoparticles for 80 minutes. Subsequently, the cells were washed and stained with serum-free medium containing 2′,7′-dichlorodihydrofluorescein diacetate (DCFH-DA) for 30 minutes at 37 °C in the dark. Images were captured using a confocal laser scanning microscope (Leica, Germany). For flow cytometric analysis, CT26 cells from the five treatment groups were harvested, resuspended in serum-free medium containing DCFH-DA, and incubated in the dark at 37 °C for 30 minutes. The mean fluorescence intensity (MFI) was then measured by flow cytometry.

### *In vitro* uptake

*In vitro* cell uptake was evaluated using CT26, HUVECs, and NCM460 cells. The cells were seeded in confocal dishes and incubated overnight individually, the combined radiotherapy group requires 2 Gy of radiotherapy to be administered after cell attachment. PLGA/DiI and Fucoidan-PLGA/DiI nanoparticles loaded with DiI dye were prepared using the method described above, the cells were co-incubated with fluorescent nanoparticles for 80 minutes, stained with DAPI to label the cell nuclei, and with DiO dye to label the cell membranes. Cell uptake was observed using a confocal laser scanning microscope. The nanoparticle uptake by CT26 and HUVECs cells was quantified using a flow cytometer, the combined radiotherapy group requires 2 Gy of radiotherapy to be administered after cell attachment. Fucoidan-PLGA/DiO nanoparticles and PLGA/DiO nanoparticles loaded with DiO dye were prepared using the same method as described above. Cells, which were seeded the day before and were in the logarithmic growth phase, were co-incubated with Fucoidan-PLGA/DiO and PLGA/DiO nanoparticles in the dark for 150 minutes. Finally, the mean fluorescence intensity (MFI) in the FITC channel was measured by flow cytometry to quantify the phagocytosis.

### Biodistribution study

CT26 cells were pre-inoculated into the right lower abdomen of BALB/c mice. Once the subcutaneous tumors reached an appropriate size, the mice were divided into four groups. PLGA/DiR or Fucoidan-PLGA/DiR nanoparticles were intravenously injected into the tail vein. Mice that required radiotherapy were given local tumor irradiation of 6 Gy 24 hours prior to nanoparticle injection. At the specified time points post-injection, the mice were anesthetized by inhalation and then scanned using the near-infrared *in vivo* imaging system/IVIS Lumina III system (PerkinElmer, Chukchi, MA, USA). 24 hours post-injection, the mice's major organ tissues and tumor tissues were collected. The *ex vivo* organs were imaged using a near-infrared imaging system to observe the distribution of the drug within the mice.

### Effect of radiation dose on P-selectin expression

Effect of radiotherapy dose on P-selectin expression in tumor and endothelial cells: CT26 and HUVECs cells were seeded 1 day in advance and treated with different doses of radiation (0, 2, 4, 6 Gy). After 24 hours, proteins were extracted and quantified using the BCA assay. After electrophoresis and transfer to PVDF membranes, the membranes were blocked with 5% skim milk and incubated overnight at 4 °C with the specified primary antibody. The washed PVDF membranes were incubated with HRP-conjugated secondary antibody at room temperature for 2 hours. Chemiluminescence signals were detected using enhanced chemiluminescence (ECL) reagents. GAPDH was used as the internal control.

Effect of radiotherapy dose on P-selectin expression in tumor tissues: when the subcutaneous tumors in mice reached an appropriate size, the tumors were irradiated with different doses of radiation. 24 hours after irradiation, the tumors from each group of mice were fixed in 4% paraformaldehyde for multiplex immunofluorescence detection. Tumor sections were stained with P-selectin antibody (Beyotime, China) and CD31 antibody (Abcam, China).

### Immune response induced by the FPG NPs

CT26 cells (1 × 10^6^) in the logarithmic growth phase were subcutaneously injected into the right lower flank of BALB/c mice. After one week, the mice were randomly divided into five groups (*n* = 3) as follows: normal saline (NS) group, PG group, FPG group, R + PG group, and R + FPG group, where “R” represents radiotherapy. Localized radiotherapy was administered to the tumor sites of the combination therapy groups on day 7 and day 14. Subsequently, treatments with NS, PG, or FPG were administered intravenously on day 8 and day 15, with a consistent GA concentration of 4 mg kg^−1^ in the nanoparticle formulations. The radiation dose was 6 Gy per fraction, delivered one day prior to the nanoparticle injections. All mice were euthanized three days after the final administration, and tumor tissues and spleens were collected from each group for flow cytometry analysis. The following monoclonal antibodies (mAbs) were used for flow cytometry: CD3-APC, CD8a-PB450, PD-1-PE/CY7, F4/80-PE, CD11b-KO525, CD86-PE/CY7, CD80-APC, and CD11c-PE (Biolegend, USA).

### *In vivo* antitumor efficacy

CT26 cells in the logarithmic growth phase were subcutaneously injected into the right lower abdomen of BALB/c mice. After 1 week, the mice were randomly divided into five groups (*n* = 5) and treated weekly with saline (NS group), PLGA/GA (PG group), Fucoidan-PLGA/GA (FPG group), PLGA/GA + radiotherapy (R + PG group), or Fucoidan-PLGA/GA + radiotherapy (R + FPG group) (GA concentration of 4 mg kg^−1^, iv; radiation dose of 6 Gy). Radiotherapy was administered 1 day prior to drug injection. Mouse body weight and tumor volume were measured every 1–2 days until the end of the experiment. Tumor-bearing mice were euthanized 3 days after the final dose. Tumor tissues from each group of mice were then collected for Ki67 immunohistochemistry, JC-1 and TUNEL multiplex immunofluorescence analysis.

### Safety studies

Tumor tissues and major organs were collected from each group of mice for hematoxylin and eosin (H&E) staining, and blood was collected from each group for biochemical analysis. Systemic toxicity was evaluated through blood biochemical markers and H&E staining of organ sections.

### Statistical analysis

Statistical analysis was performed using GraphPad Prism 10.1.2 statistical software. Independent Student's *t*-test or one-way analysis of variance (ANOVA) was used to determine differences between treatments. Data are expressed as mean ± standard deviation unless otherwise stated. Significance levels were defined as ns (not significant, *P* > 0.05), *P < 0.05, **P < 0.01, ***P < 0.001, and ****P < 0.0001 (statistical methodology were consistent with our prior study).^23^

## Results and discussion

### Preparation and characterization of Fucoidan-PLGA/GA NPs

**Fig. 1:**
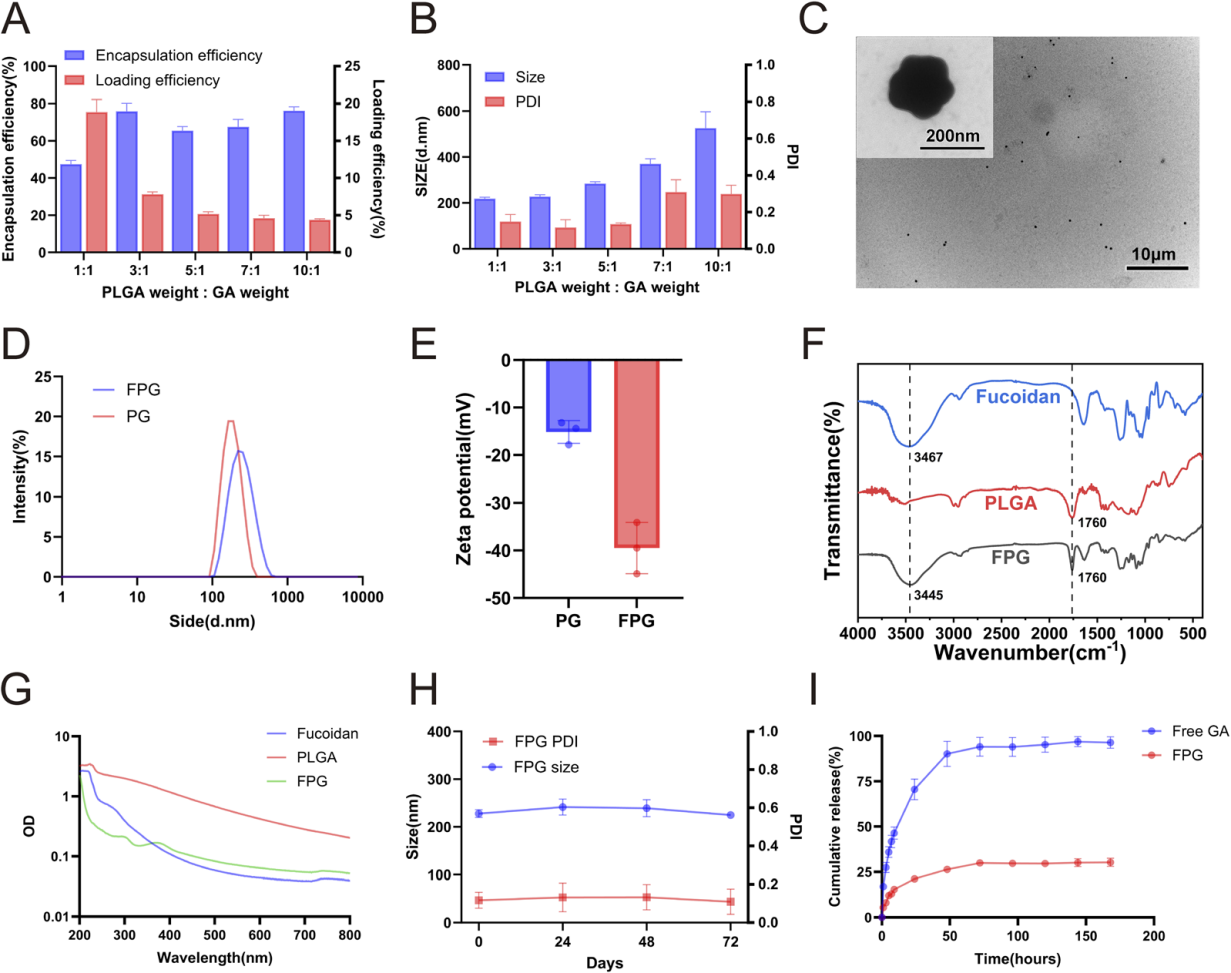
Characterization of FPG NPs. (A) The encapsulation efficiency and drug loading rate of GA in FPG with different weight ratios of PLGA and GA. Data are presented as mean ± SD (*n* = 3). (B) The size and PDI of FPG with different weight ratios of PLGA and GA. Data are presented as mean ± SD (*n* = 3). (C) The image of FPG under transmission electron microscopy (TEM) (scale bar = 200 nm, 10 µm). (D) The size distribution of PG and FPG measured by dynamic light scattering (DLS). (E) The zeta potential of PG and FPG. Data are presented as mean ± SD (*n* = 3). (F) FTIR spectroscopy of FPG, PLGA, and Fucoidan. (G) UV-vis spectroscopy of FPG, PLGA, and Fucoidan. (H) Stability monitoring of FPG in PBS over 72 hours. Data are presented as mean ± SD (*n* = 3). (I) Drug release profiles of FPG and free GA over one week. Data are presented as mean ± SD (*n* = 3).

Molecules such as CD47, CD133, FRα (folate receptor α), integrins, and MUC1 (mucin 1) can be used as tumor-specific binding targets. However, the expression of these targets seems uncontrollable, and more importantly, these nanoparticles are often unable to escape the bloodstream before reaching the tumor tissue. The strategic selection of P-selectin as a molecular target in this study was predicated on its unique pathophysiological role. Unlike other targets such as CD47 or FRα, which are often constitutively expressed, P-selectin expression is inducible and rapidly upregulated on tumor and endothelium by radiotherapy.^18,24,25^ This creates a self-amplifying targeting mechanism. Furthermore, the P-selectin/fucoidan axis mediates dynamic rolling adhesion under shear stress, which is the critical initial step for vascular extravasation,^26^ a capability not shared by many other targeting moieties. We modified fucoidan, which has a nanomolar affinity for P-selectin, on the surface of nanomicrospheres and would witness significant advantages. To explore the optimal ratio of PLGA and gambogic acid, different ratios of polylactic acid – hydroxyacetic acid copolymer (PLGA) and gambogic acid (GA) were used to prepare nanoparticles in different ratios, and the formulations with the weight ratios of 1 : 1, 3 : 1, 5 : 1, 7 : 1, and 10 : 1 (PLGA : GA) were selected for further studies, the encapsulation rate and drug loading rate of FPG NPs were calculated based on the standard curve of gambogic acid by UV spectrophotometer and the weight of each lyophilized. The results indicate that there was no positive correlation between encapsulation capacity and PLGA content (Fig. 1A). The encapsulation rate was almost the highest when the ratio of PLGA to GA was 3 : 1, and also considering that its size and PDI were also almost the smallest (Fig. 1B), the combination of PLGA and GA with a ratio of 3 : 1 was selected for the subsequent study. After determining the formulation of FPG, the fabricated nanoparticles were observed by transmission electron microscopy (Fig. 1C). The results show that the FPG nanoparticles have uniform size distribution and no aggregation. Its irregular spherical structure indicates that the hydrophilic fucoidan has successfully bound to the surface of PLGA. Furthermore, the differences in the particle size and zeta potential results also support this (Fig. 1D and E). FT-IR spectroscopy analysis showed that the spectrum of FPG nanoparticles displayed a definitive absorption peak at 1760 cm^−1^, which is the signature carbonyl (C=O) stretching vibration of the PLGA polymer. This confirms that PLGA forms the core matrix of the nanoparticles. Simultaneously, the O–H stretching vibration, which is the most prominent feature of fucoidan, was observed at 3445 cm^−1^ in the FPG spectrum. This represents a noticeable red-shift compared to its position in pure fucoidan at 3467 cm^−1^. This shift to a lower wavenumber is a classic spectroscopic indicator of the enhancement of hydrogen bonding. It strongly suggests that the hydroxyl (–OH) groups of fucoidan are engaging in new, stronger intermolecular interactions with the carbonyl (–C=O) groups of PLGA (Fig. 1F). Besides, the UV-Vis spectrum of FPG nanoparticles presents a combined spectral fingerprint of both PLGA (240 nm) and fucoidan (260 nm), serving as direct proof of the successful nanocomposite (Fig. 1G). Next, we tested the stability of FPG over 72 hours. During this period, there was no significant change in the PDI and size of the nanoparticles, indicating that FPG exhibits good stability and provides feasibility for subsequent research (Fig. 1G). We investigated the release profiles of FPG and free GA over the course of one week, measuring the drug concentration in the release medium at different time points using HPLC (Fig. 1I). The results showed that FPG did not exhibit the burst release effect observed with free GA. Over the next few days, the release rate remained at a low level, and after one week, more than 60% of the drug remained in the particles. The slow release of the drug favors the prolonged action of the nanoparticles and reduces the frequency of drug administration. Overall, the efficacy of FPG lasts for at least one week.

### *In vitro* cytotoxicity assay

**Fig. 2:**
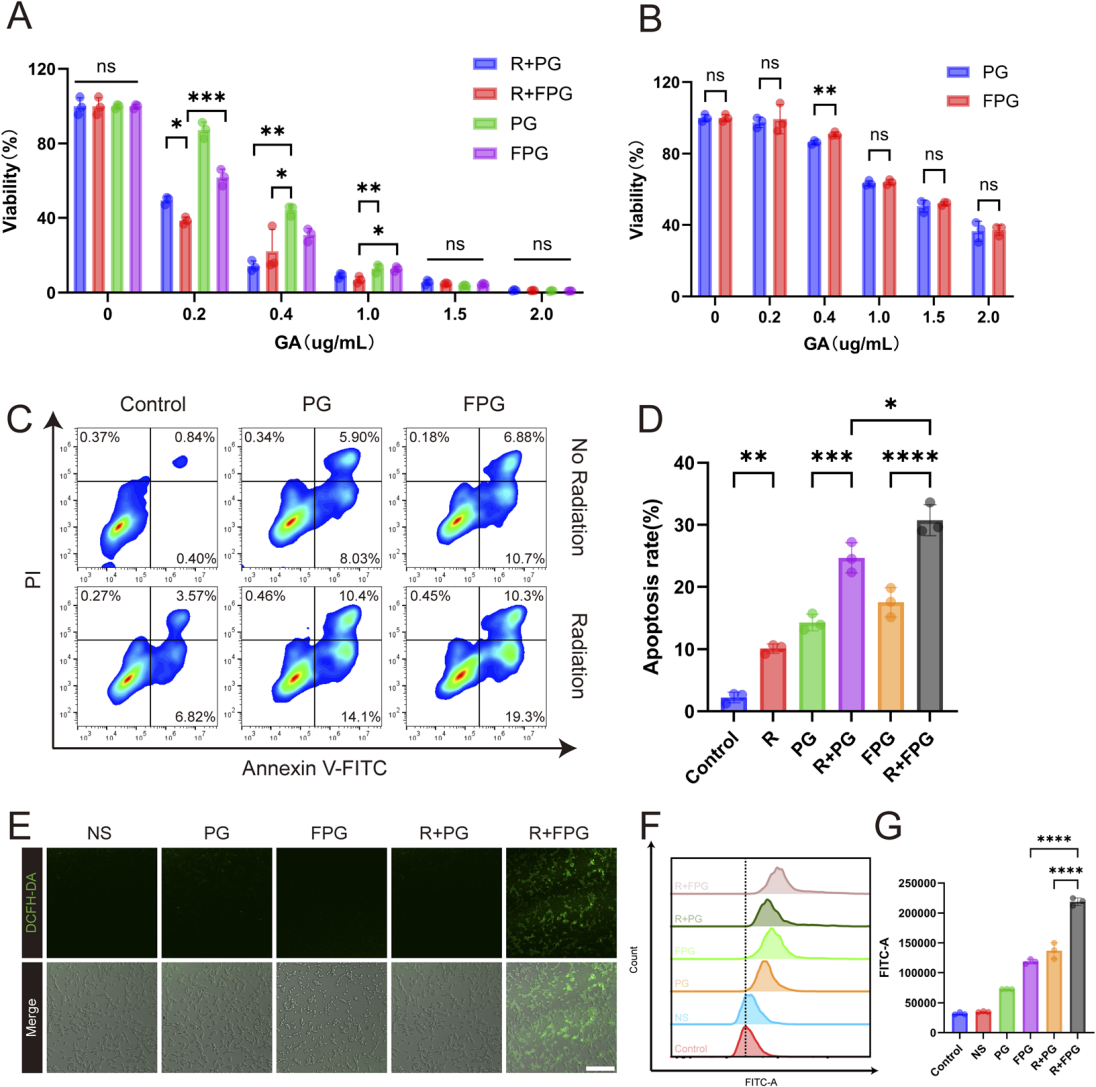
*In vitro* antitumor effects of FPG NPs. (A) Relative cell viability of CT26 cells after 24 hours of different treatments. R: Radiotherapy. Data are presented as mean ± SD (*n* = 3). *P* values were determined by one-way ANOVA followed by Tukey's multiple-comparison test. Significance levels: ***P < 0.001, ****P < 0.0001. (B) Relative cell viability of NCM460 cells after 24 hours of incubation with PG and FPG NPs. All values are expressed as mean ± SD (*n* = 3). Statistical significance between the two groups was determined by an unpaired Student's *t*-test. Significance levels: ns, *P* > 0.05; **P < 0.01. (C) Flow cytometry analysis of nanoparticle-induced apoptosis in CT26 cells. Both PG and FPG NPs contained an equivalent dose of GA (0.4 µg mL^−1^). (D) Apoptosis rate statistics of CT26 cells. R: Radiotherapy. Data are presented as mean ± SD (*n* = 3). *P* values were determined by one-way ANOVA followed by Tukey's multiple-comparison test. Significance levels: *P < 0.05, **P < 0.01, ***P < 0.001, ****P < 0.0001. (E) Confocal laser scanning microscopy (CLSM) images of CT26 cells treated with PG or FPG NPs for 80 minutes and stained with the DCFH-DA probe to monitor intracellular ROS (scale bar = 300 µm). R: Radiotherapy. (F) Flow cytometry analysis of CT26 cells under different treatments using DCFH-DA staining. R: Radiotherapy. (G) Quantification of ROS levels by flow cytometry in differently treated cells. Data are presented as mean ± SD (*n* = 3). *P* values were determined by one-way ANOVA followed by Tukey's multiple-comparison test. Significance levels: ***P < 0.001, ****P < 0.0001.

The cytotoxicity of PG and FPG on CT26 and normal human intestinal epithelial NCM460 cells was evaluated using the CCK8 assay. The results showed that after 24 hours of co-culture with CT26 cells (Fig. 2A), at a low concentration of GA (0.2 µg mL^−1^), the cell viability of the PG and FPG groups were 87.24 ± 4.34% and 61.77 ± 4.45%, respectively. In contrast, the cell viability of the R + PG and R + FPG groups decreased to 49.45 ± 2.13% and 38.54 ± 1.88%. The R + FPG group exhibited the most optimal anti-tumor effect at lower concentrations, although the differences between the groups gradually diminished as the dosage increased. These results suggest that the P-selectin targeting strategy, especially at low drug concentrations, has significant potential in enhancing tumor cell killing and radiosensitization. Notably, the cytotoxicity of FPG in NCM460 cells was not significantly different from that of PG at any concentration, indicating that the fucoidan modification did not confer additional toxicity to the normal cells (Fig. 2B). Moreover, the substantially lower cytotoxicity in NCM460 cells compared to CT26 cells (Fig. 2A) suggests a promising therapeutic window for the FPG nanoparticles.

### Apoptosis assay

The induction of apoptosis in CT26 cells by radiotherapy combined with nanoparticles was assessed using the Annexin V-FITC/PI dual staining method (Fig. 2C). The results showed that under non-radiotherapy conditions, the apoptosis rates of the PG and FPG groups were 14.25 ± 1.31% and 17.50 ± 2.37%, respectively. However, in the combined radiotherapy group (R + FPG), the apoptosis rate significantly increased to 30.74 ± 2.49% (Fig. 2D), representing a 75.7% increase relative to the FPG group and a 24.5% increase relative to the R + PG group. FPG nanoparticles, in combination with radiotherapy, exhibited a stronger pro-apoptotic effect, which may be related to their targeting of P-selectin, enhancing tumor cell uptake efficiency. In addition to inducing tumor cell apoptosis,^27,28^ the action of gambogic acid at the tumor site may also be associated with its anti-angiogenic effects,^29,30^ cause tumor cell cycle arrest,^31^ induce autophagy in tumor cells,^32^ downregulate EMT to inhibit tumor metastasis and invasion,^33^ and limit the expression of MDM2 to stabilize and activate the tumor suppressor gene p53,^34^ among other mechanisms.

### ROS measurement

Confocal microscopy images revealed that the FPG combination radiotherapy group exhibited more intense green fluorescence, demonstrating a significant increase in intracellular ROS levels (Fig. 2E). This finding was corroborated by quantitative data from flow cytometry, which showed that the ROS levels in the R + FPG group were 84.76% and 59.98% higher than those in the FPG group and the R + PG group, respectively (Fig. 2F and G). Following this treatment, tumor cells subsequently experienced a sharp surge in ROS. The elevated ROS levels attacked biological macromolecules such as DNA, proteins, and lipids, inducing oxidative stress damage and leading to an irreversible outcome—apoptosis. This ROS-dependent apoptotic pathway is recognized as a common mechanism of action for a number of antitumor compounds, including gambogic acid.^35,36^

### *In vitro* uptake

**Fig. 3:**
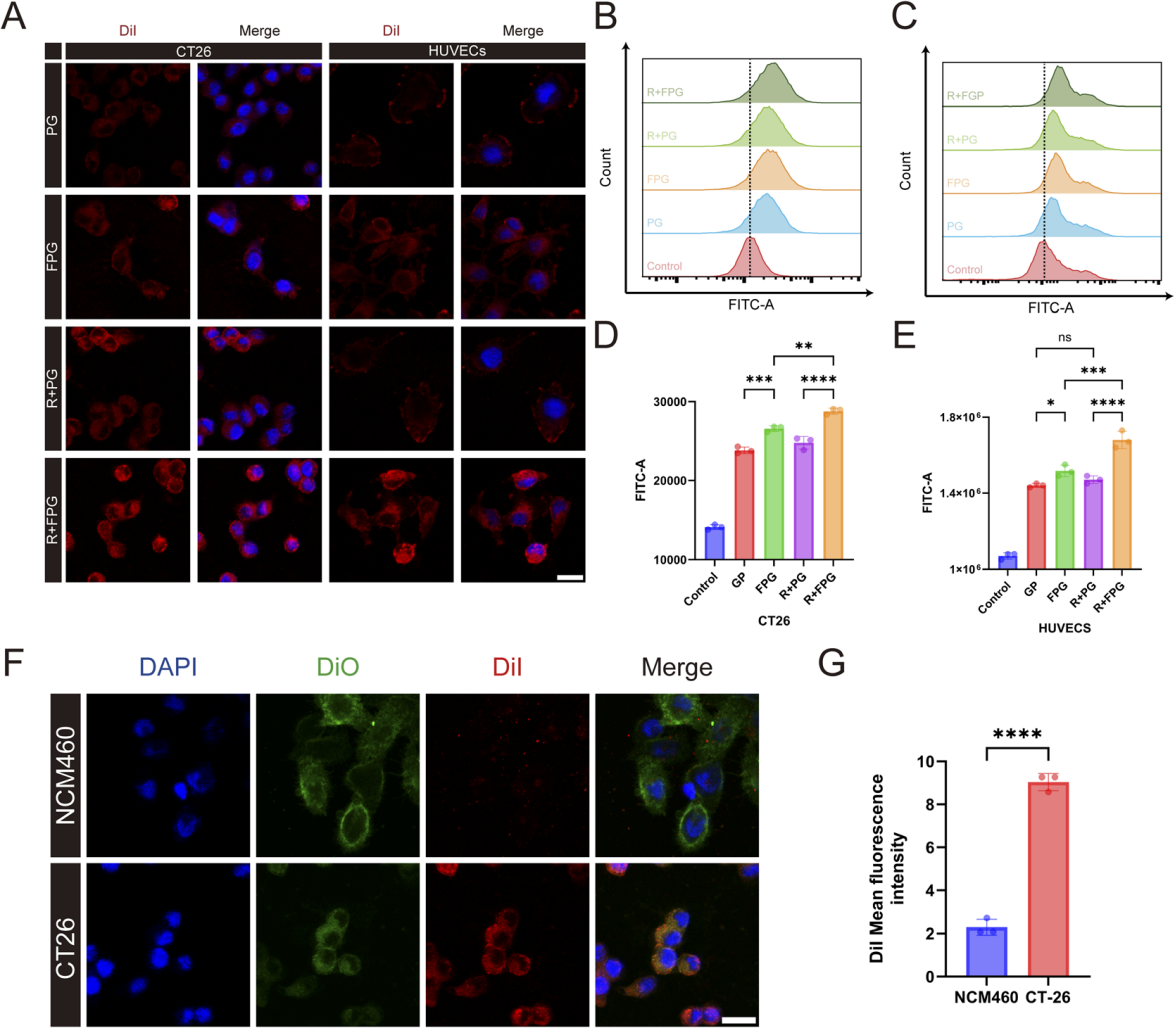
Cellular uptake of nanoparticles. (A) Confocal images of nanoparticles co-incubated with CT26 and HUVECs cells. R: Radiotherapy. Nanoparticles were labeled with DiI, and cell nuclei were stained with DAPI (scale bar = 20 µm). (B) Representative flow cytometry plots of CT26 cell uptake of nanoparticles, where NPs were labeled with DiO. (C) Representative flow cytometry plots of HUVECs cell uptake of nanoparticles. NPs were labeled with DiO. (D) Flow cytometry analysis of the mean fluorescence intensity (MFI) of CT26 cells. Data are presented as mean ± SD (*n* = 3). *P* values were determined by one-way ANOVA followed by Tukey's multiple-comparison test. Significance levels: **P < 0.01, ***P < 0.001, ****P < 0.0001. (E) Flow cytometry analysis of the mean fluorescence intensity (MFI) of HUVECs cells. Data are presented as mean ± SD (*n* = 3). *P* values were determined by one-way ANOVA followed by Tukey's multiple-comparison test. Significance levels: ns, *P* > 0.05; *P < 0.05, ***P < 0.001, ****P < 0.0001. (F) Confocal images of FPG uptake by CT26 and NCM460 cells. NPs were labeled with DiI, cell membranes with DiO, and cell nuclei were stained with DAPI (scale bar = 20 µm). (G) Quantitative analysis of FPG uptake by CT26 and NCM460 cells. All values are expressed as mean ± SD (*n* = 3). Statistical significance between the two groups was determined by an unpaired Student's *t*-test. Significance levels: ****P < 0.0001.

Fucoidan, a natural ligand of P-selectin, was used in this experiment as a surface modifier of FPG to bind with the P-selectin overexpressed on the tumor tissue surface, demonstrating excellent targeting ability when combined with radiotherapy induction. After co-culturing CT26 and HUVEC cells with PLGA/DiI and Fucoidan-PLGA/DiI nanoparticles, the cells were observed using a confocal microscope. The results showed that the R + FPG group of both cell types exhibited the highest red signal (DiI dye labeling the nanoparticle core) among all groups, and DAPI (blue) was used to stain the nuclei. The presence of intense red fluorescence in the cytoplasm of both CT26 cells and HUVECs, showing a characteristic perinuclear distribution, is consistent with the effective internalization of the nanoparticles (Fig. 3A). The FPG content in the radiotherapy-treated cells was higher than in the other three groups, indicating that radiotherapy can enhance the uptake of FPG by the cells.

CT26 and HUVEC cells were co-cultured with two types of nanoparticles labeled with DiO dye (PLGA/DiO NPs and Fucoidan-PLGA/DiO NPs), and the mean fluorescence intensity (MFI) in the FITC channel was measured using flow cytometry (Fig. 3B–E). Quantitative analysis revealed that both cell types exhibited the highest phagocytic uptake in the radiotherapy combined with Fucoidan-PLGA/DiO NPs group, with the mean fluorescence intensity (MFI) of the R + FPG group being 17.7% higher (**P < 0.01) in CT26 cells and 36.6% higher (***P < 0.001) in HUVEC cells compared to the control FPG group. No significant difference was observed in the PG group. This also indicates that radiotherapy, by inducing P-selectin overexpression, significantly enhanced the phagocytic efficiency of tumor and endothelial cells through the mediated endocytic pathway, thereby achieving a truly dual-targeting strategy. Fucoidan-PLGA/GA NPs can not only target P-selectin on the surface of tumor cells but also enhance the ability to cross the vascular barrier by binding to P-selectin on tumor vascular endothelial cells. Similar to the experimental results of Daniel E. Tylawsky, fucoidan-modified nanoparticles targeting the tumor vasculature facilitate caveolin-1-dependent transendothelial transport, and it was demonstrated that this transcytosis-mediated process induces active crossing of the blood–brain barrier in a P-selectin-dependent manner.^37^

NCM460 normal intestinal epithelial cells and CT26 colorectal cancer cells were selected to evaluate the uptake differences of Fucoidan-PLGA/GA NPs (DiI was used to label the nanoparticles, DiO green fluorescence dye labeled the cell membranes of both cell types, and DAPI was used to stain the cell nuclei). We observed that compared to CT26 cells, NCM460 cells exhibited a lower uptake efficiency of Fucoidan-PLGA/GA NPs, indirectly demonstrating that the nanoparticles have relative safety and low biological toxicity (Fig. 3F and G).

### Biodistribution study

**Fig. 4:**
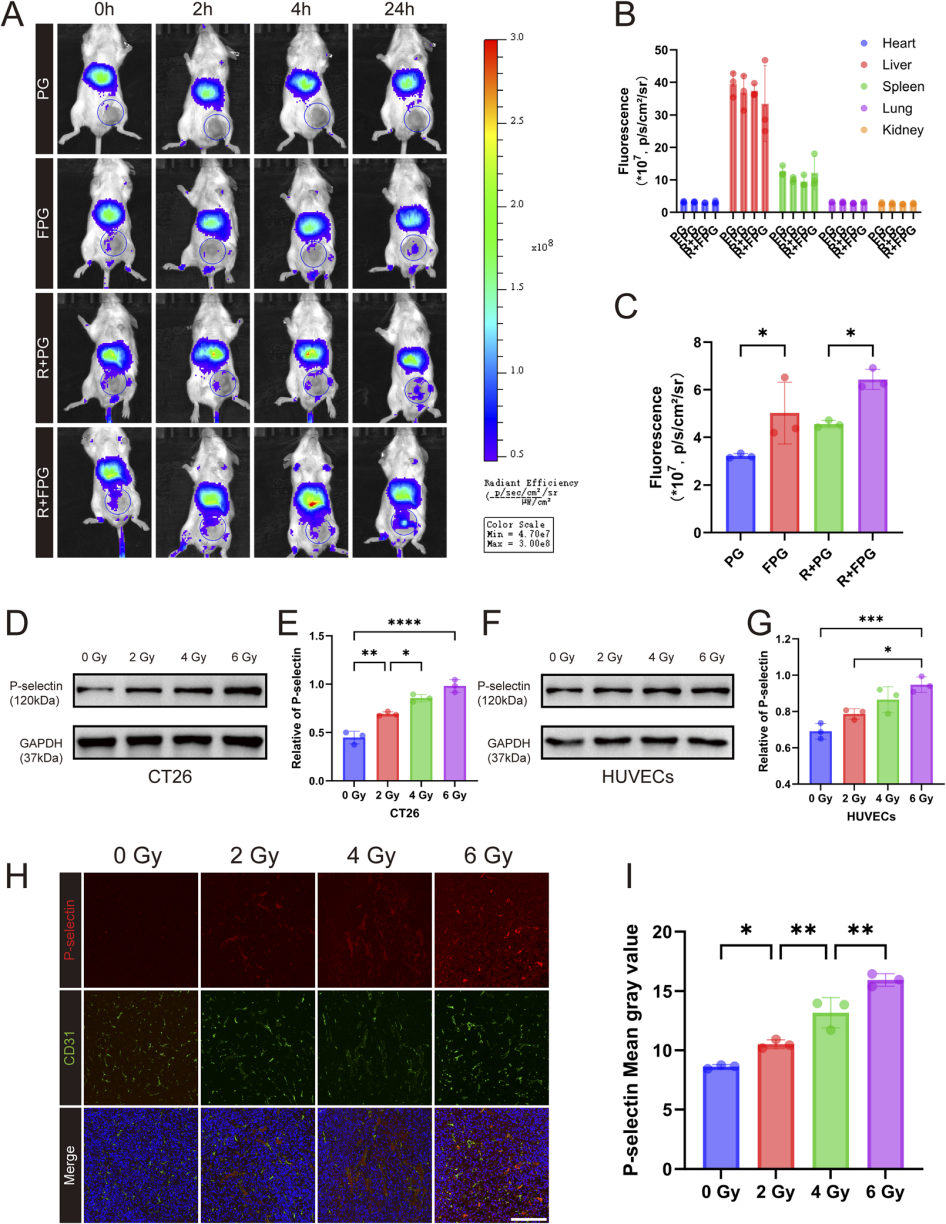
Biodistribution of nanoparticles and expression of P-selectin. (A) Fluorescence imaging of mice after injection of fluorescent nanoparticles, with nanoparticles labeled with the fluorescent dye DIR. R: Radiotherapy. (B) Fluorescence intensity of the mouse's internal organs. Error bars represent mean ± SD (*n* = 3). (C) Fluorescence intensity of the tumor only. Data are presented as mean ± SD (*n* = 3). *P* values were determined by one-way ANOVA followed by Tukey's multiple-comparison test. Significance levels: *P < 0.05. (D) and (F) Effect of radiotherapy dose on P-selectin protein expression in CT26 and HUVECs cells. (E) and (G) Quantitative analysis of P-selectin protein expression in CT26 and HUVECs cells. Data are presented as mean ± SD (*n* = 3). *P* values were determined by one-way ANOVA followed by Tukey's multiple-comparison test. Significance levels: *P < 0.05, **P < 0.01, ***P < 0.001, ****P < 0.0001. (H) Effect of radiotherapy dose on P-selectin expression in tumor tissue (scale bar = 200 µm). (I) Quantitative analysis of P-selectin expression in tumor tissue after radiotherapy dose. Data are presented as mean ± SD (*n* = 3). *P* values were determined by one-way ANOVA followed by Tukey's multiple-comparison test. Significance levels: *P < 0.05, **P < 0.01.

Radiotherapy, a commonly used method for inhibiting the progression of malignant tumors, is highly effective in guiding nanoparticles to target specific tumor regions.^38^ Radiation can be precisely and selectively delivered to the tumor while protecting surrounding non-malignant tissues.^39,40^ To differentiate the permeability increase caused by radiation-induced vascular damage, we also included control nanoparticles. The biodistribution of NPs was detected using a near-infrared fluorescence imaging system, with GA being replaced by DIR. Twenty-four hours after intravenous injection, the tumor region of the FPG group in CT26 tumor-bearing mice with radiation pre-treatment exhibited bright blue fluorescence, which was higher than that of the other three control groups. No significant differences were observed between the four groups in the *ex vivo* organ fluorescence (Fig. 4A–C). In the drug distribution experiment, P-selectin-targeted nanoparticles showed significantly higher drug accumulation in the tumor region compared to the control group. The fluorescence in the tumor region of the PG group after irradiation also slightly increased, which is likely a normal phenomenon due to radiation-induced endothelial cell damage and increased permeability.^41^

### Effect of radiation dose on P-selectin expression

Recent studies have found that P-selectin is overexpressed on cancer cells and tumor-associated blood vessels in many human cancers.^18,24,37^ Furthermore, local radiation induction in tumors with personalized expression differences can achieve levels much higher than in normal tissues.^18^ We assessed the protein expression levels of P-selectin in CT26 and HUVEC cells after treatment with different radiation doses. The results showed that within a certain range, the expression levels of P-selectin in both cell types increased to varying degrees with the radiation dose, and this upregulation was dose-dependent, which is consistent with the theory presented earlier (Fig. 4D–G).

Multiple immunofluorescence was used to detect changes in P-selectin and the endothelial cell marker CD31 in tumor tissues after irradiation. Mice were exposed to different radiation doses, and tissues were collected 24 hours post-irradiation. It was found that the expression of P-selectin in tumor tissues also increased in a dose-dependent manner (Fig. 4H–I), which is consistent with the results of the western blot experiments conducted *in vitro*.

### Immune response induced by the FPG NPs

**Fig. 5:**
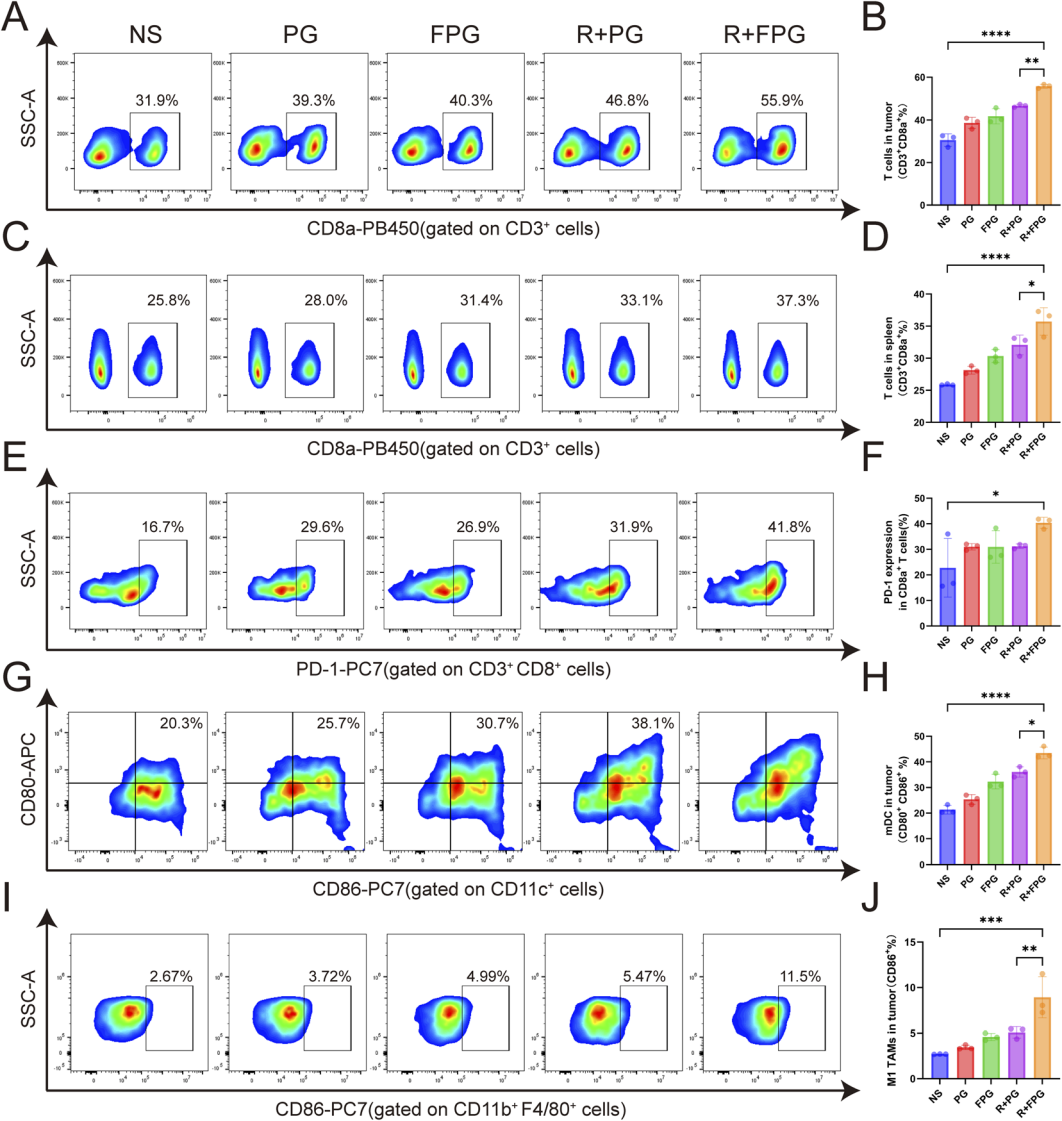
Immune response induced by the FPG NPs. (A) Representative flow cytometry plots of CD3^+^ CD8a^+^ T cells from tumor tissues in each treatment group. (B) Quantification of CD3^+^ CD8a^+^ T cells in mouse tumors across treatment groups. Data are presented as mean ± SD (*n* = 3). *P* values were determined by one-way ANOVA followed by Tukey's multiple-comparison test. Significance levels: **P < 0.01, ****P < 0.0001. (C) Representative flow cytometry plots of CD3^+^ CD8a^+^ T cells from mouse spleens in each treatment group. (D) Quantification of splenic CD3^+^ CD8a^+^ T cells across treatment groups. Data are presented as mean ± SD (*n* = 3). *P* values were determined by one-way ANOVA followed by Tukey's multiple-comparison test. Significance levels: *P < 0.05, ****P < 0.0001. (E) Representative flow cytometry plots showing PD-1 expression on tumor-infiltrating CD3^+^ CD8a^+^ T cells. (F) Quantification of PD-1 expression by flow cytometry. Data are presented as mean ± SD (*n* = 3). *P* values were determined by one-way ANOVA followed by Tukey's multiple-comparison test. Significance levels: *P < 0.05. (G) Representative flow cytometry plots of CD80^+^ CD86^+^ mDCs from mouse tumors in each treatment group. (H) Quantification of CD80^+^ CD86^+^ mDCs by flow cytometry. Data are presented as mean ± SD (*n* = 3). *P* values were determined by one-way ANOVA followed by Tukey's multiple-comparison test. Significance levels: *P < 0.05, ****P < 0.0001. (I) Representative flow cytometry plots of CD11b^+^ F4/80^+^ CD86^+^ TAMs (M1-type) from mouse tumors in each treatment group. (J) Quantification of M1-type tumor-associated macrophages (M1 TAMs) by flow cytometry. Data are presented as mean ± SD (*n* = 3). *P* values were determined by one-way ANOVA followed by Tukey's multiple-comparison test. Significance levels: **P < 0.01, ***P < 0.001.

The immunomodulatory effects of the FPG NPs and radiotherapy combination were assessed *in vivo via* flow cytometric analysis of tumor and splenic tissues. The results revealed that this combination strategy potently enhanced anti-tumor immunity. Specifically, it led to the highest proportions of CD3^+^ CD8a^+^ T cells in both the tumor (55.7%) and spleen (35.7%) among all groups (Fig. 5A–D). Strikingly, the combination therapy doubled the proportion of mature dendritic cells (CD11c^+^ CD80^+^ CD86^+^) in tumors (43.5%) compared to the NS control (20.3%) (Fig. 5G and H). Furthermore, and in line with the known immunostimulatory function of gambogic acid,^42^ we found a significant increase in M1-type tumor-associated macrophages (CD11b^+^ F4/80^+^ CD86^+^) (Fig. 5I and J). Notably, the tumor-infiltrating CD8a^+^ T cells also exhibited increased PD-1 expression (Fig. 5E and F). These findings indicate that the treatment successfully remodeled the tumor immune landscape, converting it from a “cold” to a “hot” state. The observed PD-1 upregulation points to a potential feedback mechanism and suggests that co-targeting this pathway could be a promising future direction.

### *In vivo* antitumor efficacy

**Fig. 6:**
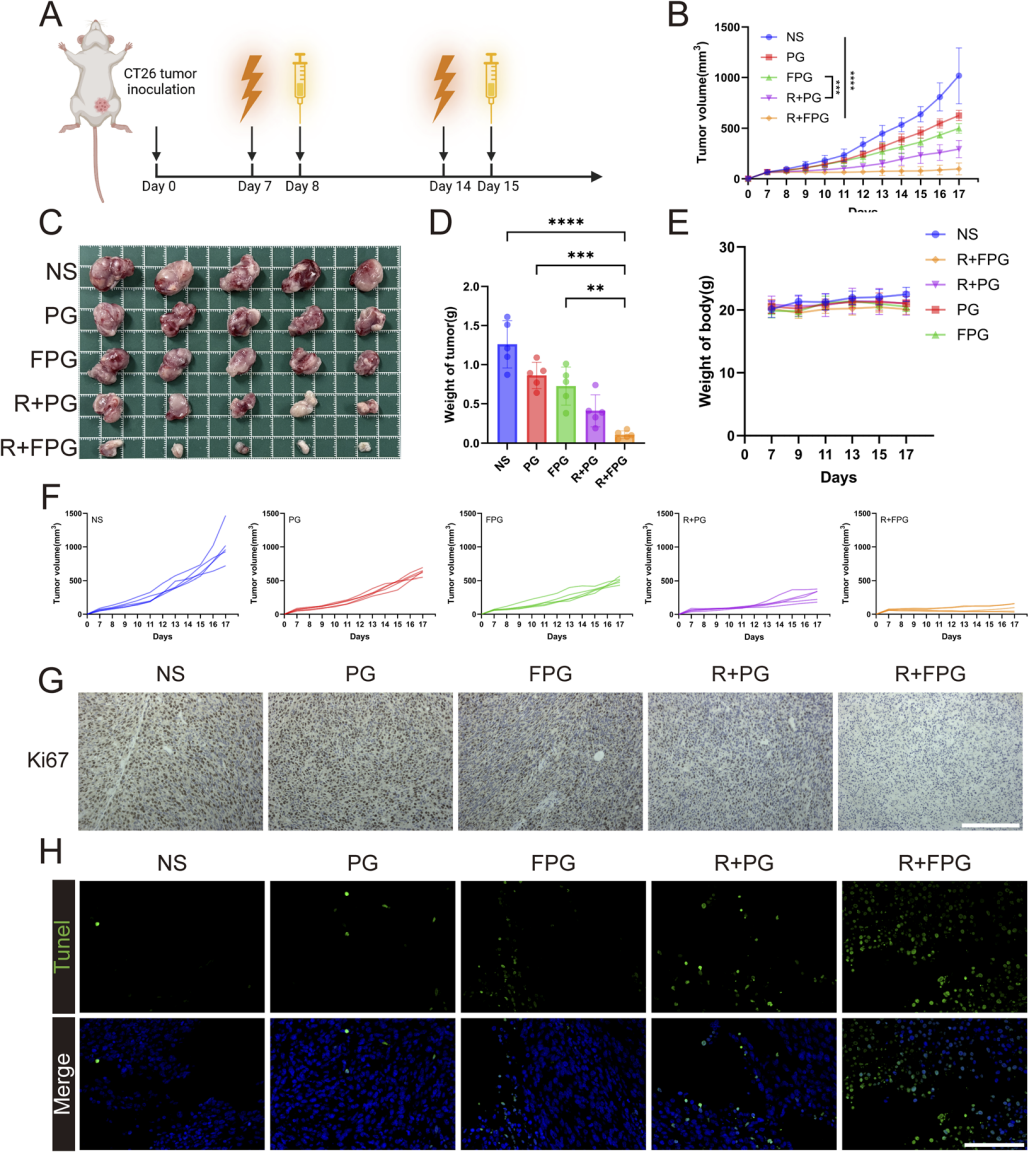
Evaluation of the antitumor efficacy of FPG NPs *in vivo*. (A) Diagram of CT26 tumor inoculation and treatment schedule. Tumors were inoculated on day 0, with radiotherapy administered on days 7 and 14, and intravenous injections of the drug on days 8 and 15. (B) Tumor growth curves for each group. Error bars represent mean ± SD (*n* = 5). *P*-values were calculated using two-way ANOVA; *** indicates *P* < 0.001, **** indicates *P* < 0.0001. (C) Photographs of tumors from each group after treatment. (D) Tumor weight from each group after treatment. Data are presented as mean ± SD (*n* = 3). *P* values were determined by one-way ANOVA followed by Tukey's multiple-comparison test. Significance levels: **P < 0.01, ***P < 0.001, ****P < 0.0001. (E) Changes in body weight of mice from each group during treatment. Error bars represent mean ± SD (*n* = 5). (F) Tumor growth curves of mice from each group during treatment. (G) Ki67 staining of tumor tissue (scale bar = 200 µm). (H) Representative images of TUNEL immunofluorescence staining of tumor sections (scale bar = 100 µm).

**Fig. 7:**
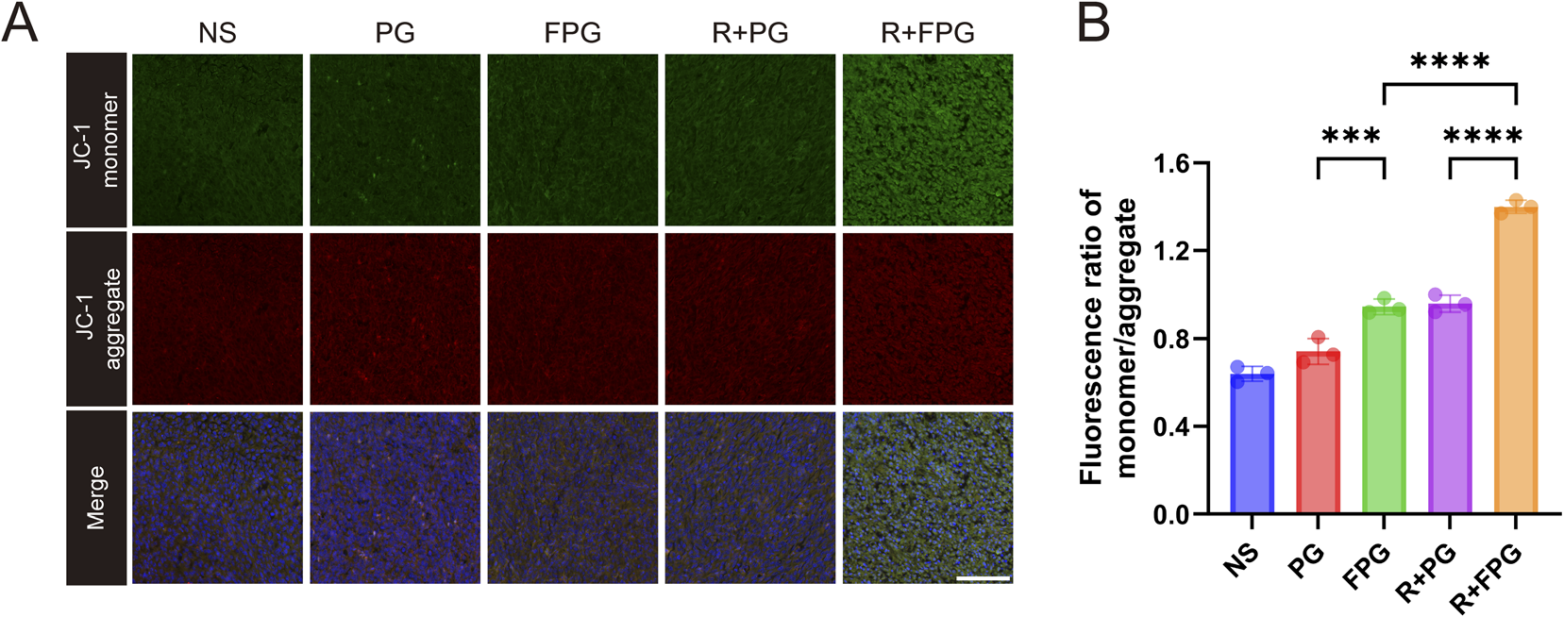
JC-1 staining. (A) Representative JC-1 immunofluorescence images of tumor sections from each of the five treatment groups (scale bar = 100 µm). (B) Analysis of the mean fluorescence ratio of green (monomer)/red (aggregate) intensity of JC1 staining. Data are presented as mean ± SD (*n* = 3). *P* values were determined by one-way ANOVA followed by Tukey's multiple-comparison test. Significance levels: ***P < 0.001, ****P < 0.0001.

The limitations and singularity of traditional nanoparticle drug delivery, combined with the phenomenon of drug “passing through without staying” due to the endothelial barrier, have hindered current cancer treatments. Precise drug delivery to the lesion area can significantly improve therapeutic efficacy, and nanoparticles for solid tumors must break through the tumor vasculature in order to increase drug infiltration in the tumor mesenchyme.^43^ We attempted *in vivo* experiments combining FPG with radiotherapy. CT26 tumor-bearing BALB/C mice were divided into five groups (*n* = 5) for treatment: the NS group, PG group, FPG group, R + PG group, and R + FPG group. The mice were treated according to the protocol outlined in the flowchart (Fig. 6A). The results of the tumor growth curve indicate that (Fig. 6B–D), compared to the other four groups, only two cycles of R + FPG treatment achieved sustained stability in all tumors within the group for 10 days. The tumor inhibition rate reached 90.36%, significantly higher than the 38.39% in the PG group, 51.01% in the FPG group, and 71.10% in the R + PG group. In all treatment groups, mice treated with FPG NPs combined with radiotherapy demonstrated significant resistance to tumor progression. These experimental results confirm the excellent anti-tumor capability of this specifically targeted nanoparticle combined with local tumor radiotherapy. Immunohistochemical analysis of tumor tissues showed that the expression level of the proliferation-related protein Ki-67 was significantly decreased after treatment in the experimental group (Fig. 6G). Immunofluorescence analysis of tumor tissues demonstrated that the combination of FPG nanoparticles and radiotherapy effectively activated the mitochondria-mediated intrinsic apoptotic pathway. JC-1 staining revealed an early apoptotic event—the collapse of mitochondrial membrane potential, as evidenced by an increase in the green-to-red fluorescence intensity ratio (Fig. 7A and B). In contrast, TUNEL staining captured the irreversible stage of apoptosis—extensive DNA fragmentation, manifested as numerous TUNEL-positive nuclei (Fig. 6H). These complementary findings from distinct subcellular locations (mitochondria *vs.* nucleus) and different phases of apoptosis (early *vs.* late) mutually corroborate each other, collectively affirming the efficacy of this combinatory treatment in triggering programmed cell death in tumor cells.

The therapeutic effects of gambogic acid at the tumor site are not only due to direct cellular effects but also involve the reprogramming of the immune microenvironment. In previous reports, we found that gambogic acid can increase the proportion of M1 macrophages^42^ and promote dendritic cell (DC) maturation, thereby further activating the expression of CD8^+^ T cells.^44^ Our flow cytometry data on the immune microenvironment also support this notion. Similarly, radiation, as an acute injury, also induces acute inflammation and triggers complex responses in the tumor microenvironment (TME), leading to immune effects in the tumor.^45^ The excessive production of local inflammation and chemokines leads to increased infiltration of immune cells and T lymphocytes. By inducing immunogenic cell death (ICD), new antigens are generated in tumor cells.^46^ Radiation enhances the maturation of dendritic cells (DCs) and their antigen-presenting capacity.^47^ These effects, combined with the excellent dual anti-tumor activity of gambogic acid, may together establish a robust tumor-suppressive immune microenvironment. The development and treatment of tumors is a systematic and complex process. In the future, more comprehensive experimental validation will be required based on this foundation.

### Safety studies

**Fig. 8:**
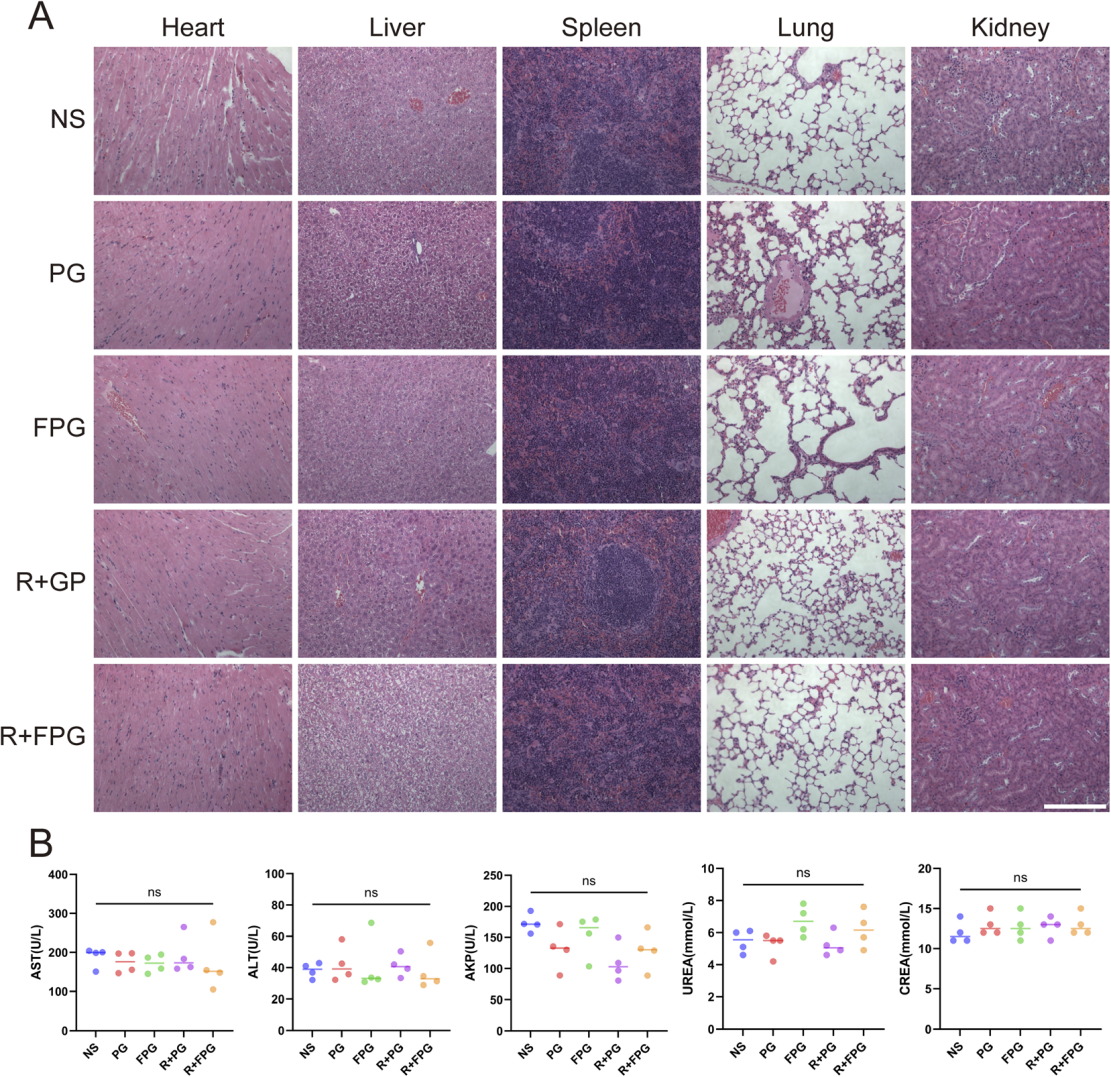
Biosafety assessment of FPG NPs *in vivo* (A) H&E staining images of major organs from each group of mice after treatment (scale bar = 200 µm). (B) Blood biochemical test results from each group of mice after treatment (*n* = 4). AST, aspartate aminotransferase; ALT, alanine aminotransferase; AKP, alkaline phosphatase; UREA, blood urea nitrogen; CREA, serum creatinine. *P*-values were calculated using one-way ANOVA and Tukey's multiple comparisons test; ns indicates *P* > 0.05.

Compared to traditional gambogic acid microspheres, the hydrophilic polysaccharide not only increases the stability of the nanoparticle structure,^48^ but also demonstrates excellent biocompatibility. During the treatment, no significant weight loss was observed in any of the mouse groups. Mice in the NS group showed a slow and steady weight gain throughout the period, while the remaining four groups exhibited a transient weight loss after two treatments, which quickly returned to normal. Such minor fluctuations are within the acceptable range during the treatment process (Fig. 6E). Additionally, the organ histology images of all groups showed no significant differences or signs of organ damage (Fig. 8A). No significant differences (*P* > 0.05) were observed in the biochemical markers of the mice's blood across the five treatment groups, all of which remained within the normal range (Fig. 8B). These results conclusively demonstrate that FPG combined with radiotherapy, at the treatment dose, has no significant impact on the health of the mice.

## Conclusions

**Fig. 9:**
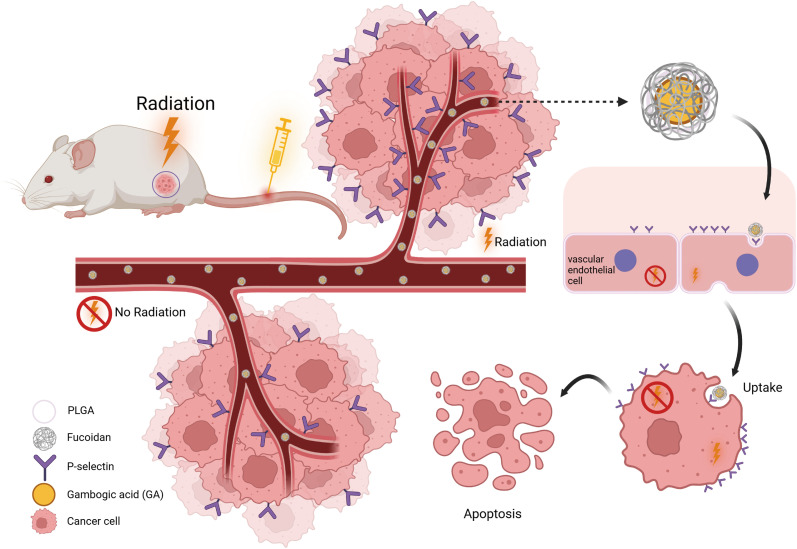
Radiotherapy-induced expression of P-selectin, which subsequently promotes the targeted delivery of FPG.

In conclusion, we successfully designed and constructed P-selectin-targeted gambogic acid nanoparticles, which enhance therapeutic effects through radiation preexcited (Fig. 9). Furthermore, the combination of FPG and radiotherapy demonstrates significant advantages in tumor cell uptake, cytotoxicity, induction of apoptosis, and modulating the immune microenvironment. Combination therapy not only improved treatment efficiency but also ensured the safety of the drug *in vivo*. This precision treatment for malignant tumors successfully delivers the drug to the tumor site through the endothelial barrier under external guidance, while also promoting drug entry into the tumor tissue through P-selectin-dependent endocytosis, thereby inhibiting tumor progression. Combined with the anti-tumor effects of radiotherapy itself, it provides a multi-pronged attack on tumor cells. This spatiotemporally controlled combination therapy has significant potential for clinical translation and offers a novel strategy for the treatment of colorectal cancer.

## Ethical statement

All animal experiments were approved by the Laboratory Animal Care and Use Committee of the Affiliated Nanjing Drum Tower Hospital of Nanjing University Medical School (Checking number: 2024AE01080), and were carried out in compliance with all relevant ethical regulations.

## Author contributions

Liuqi Sang and Qun Zhang led the project, designed and performed the experiments, analyzed the data, and wrote the initial manuscript. Zixin Liang assisted in experimentation and analysis. Xinyi Xu, Xueying Bai, Ning Wang, Xingzhi Han, and Li Li conducted the research. Jing Hu provided conceptual guidance and manuscript revisions. Xiaoping Qian secured funding, provided overall supervision, and critically revised the manuscript.

## Conflicts of interest

The authors affirm that there are no financial or non-financial competing interests associated with this work.

## Supplementary Material

RA-015-D5RA02815A-s001

RA-015-D5RA02815A-s002

## Data Availability

The data that support the findings of this study are not publicly available due to privacy restrictions. However, they can be obtained from the corresponding author upon reasonable request. Supplementary information (SI) is available. See DOI: https://doi.org/10.1039/d5ra02815a.
