# Supplementary figures and images for: Application of radiotherapy-preexcited gambogic acid dual targeting nanoparticles in colorectal cancer
Source: RSC Adv. 2025 Dec 3;15(55):47506–19. doi: 10.1039/d5ra02815a (PMC12679429; doi:10.1039/d5ra02815a)

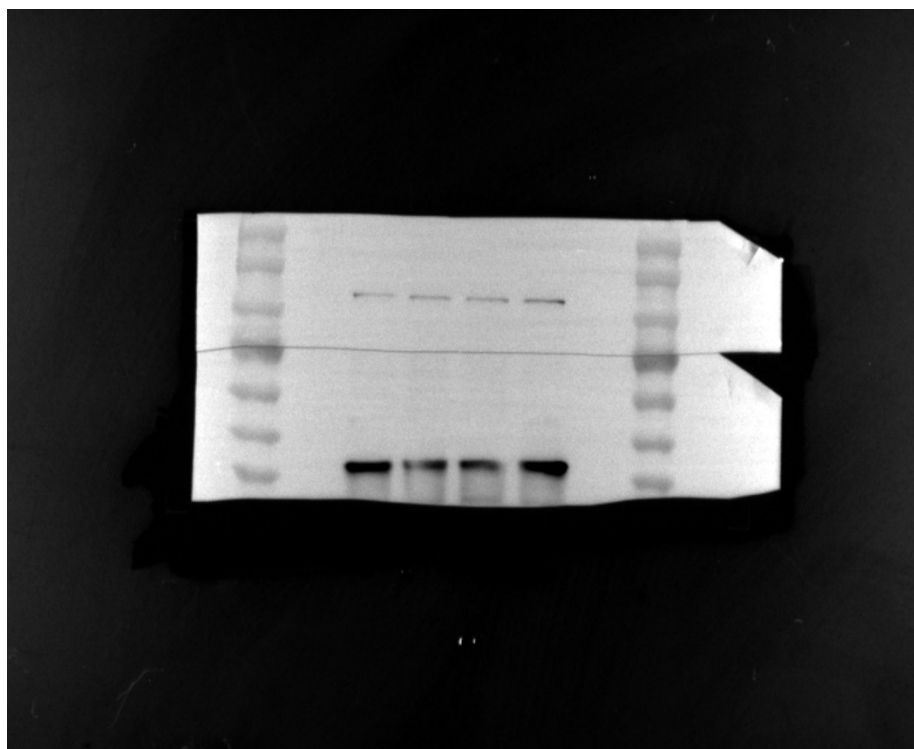

CT26 all

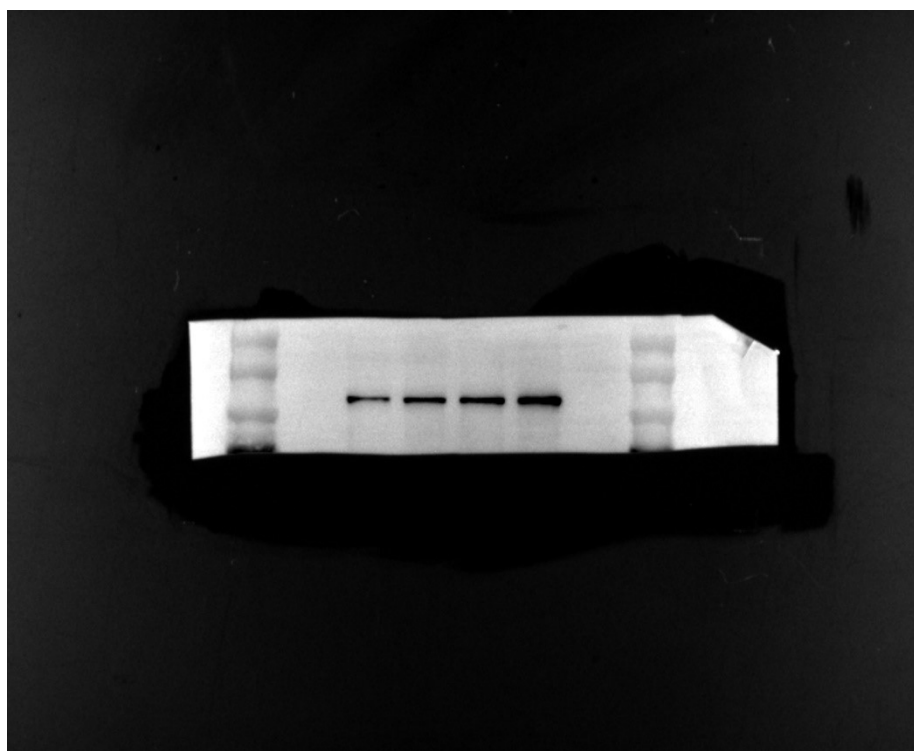

CT26 CD62P

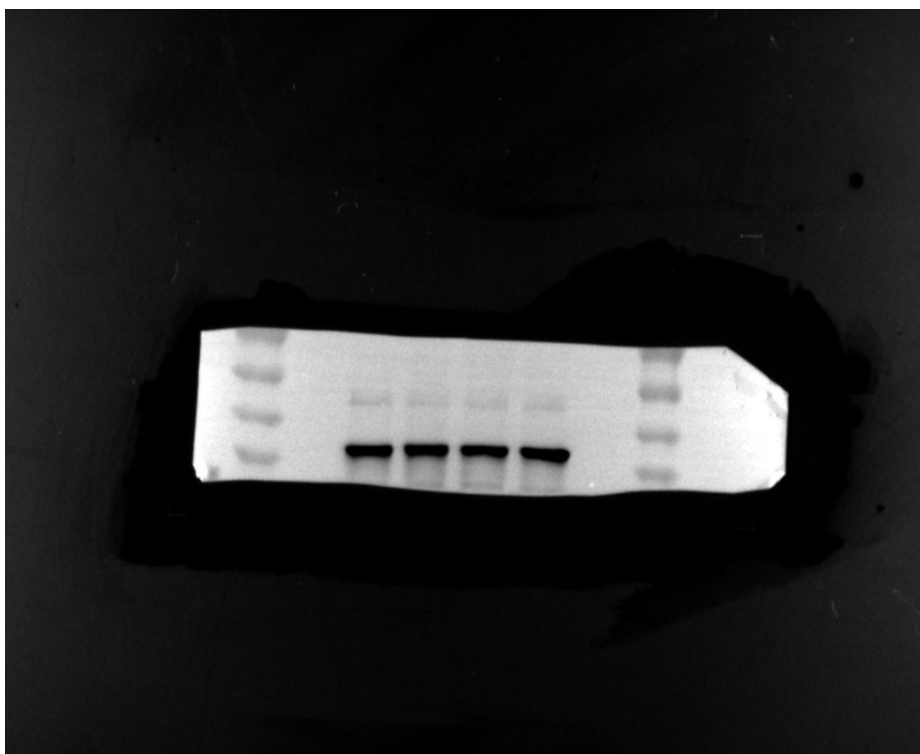

CT26 GADPH

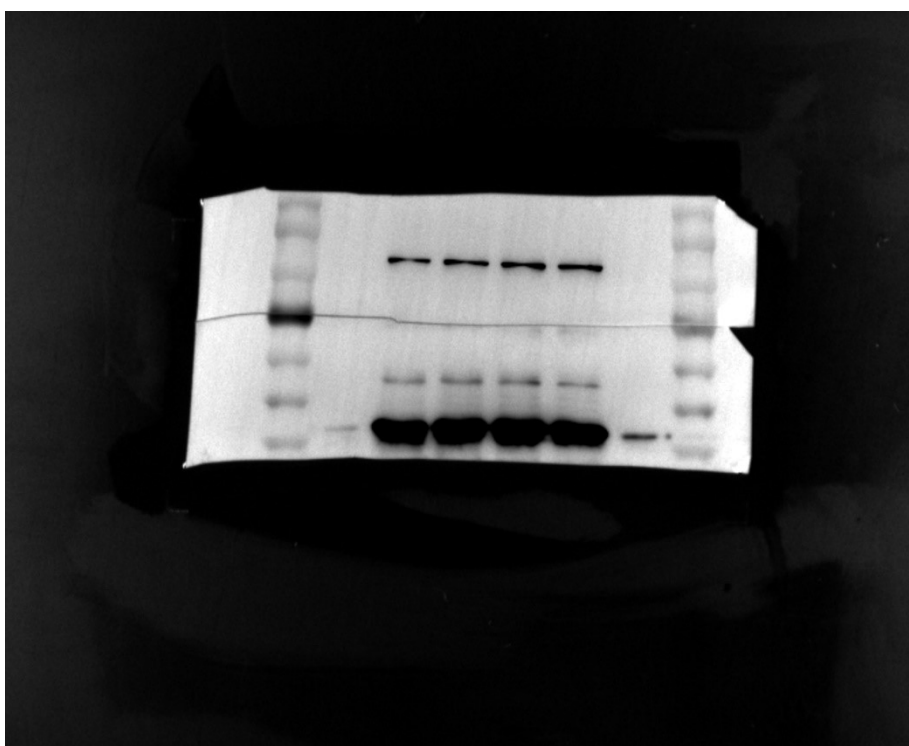

Huvecs all

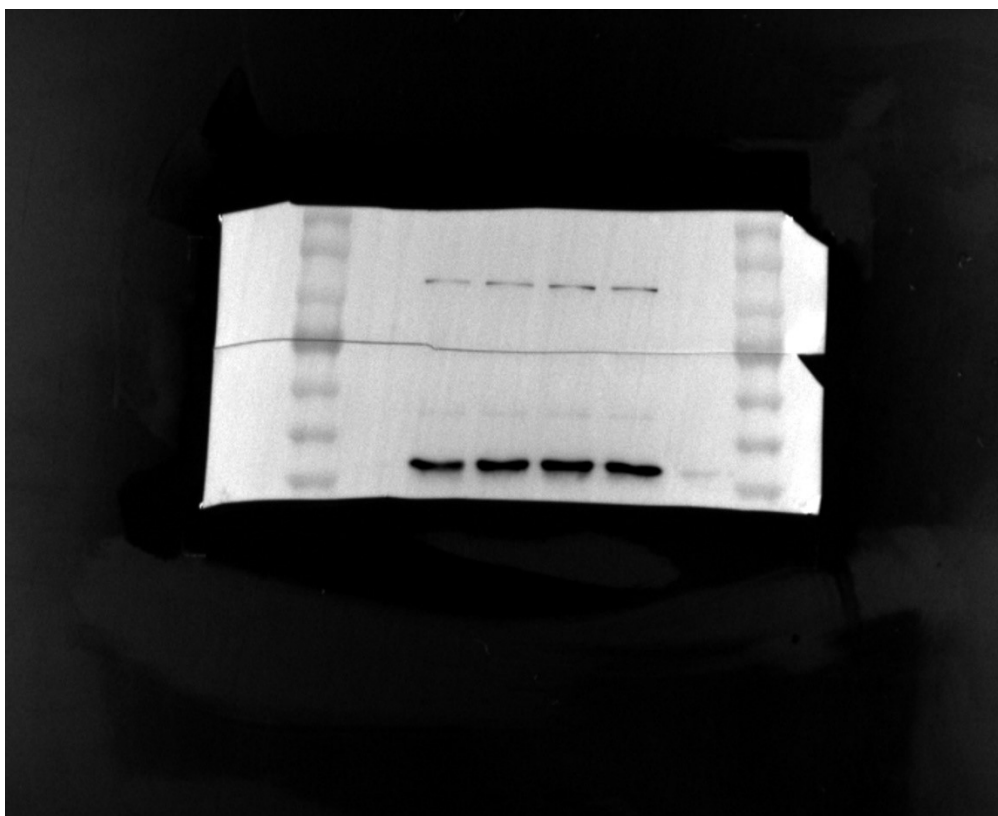

Huvecs all

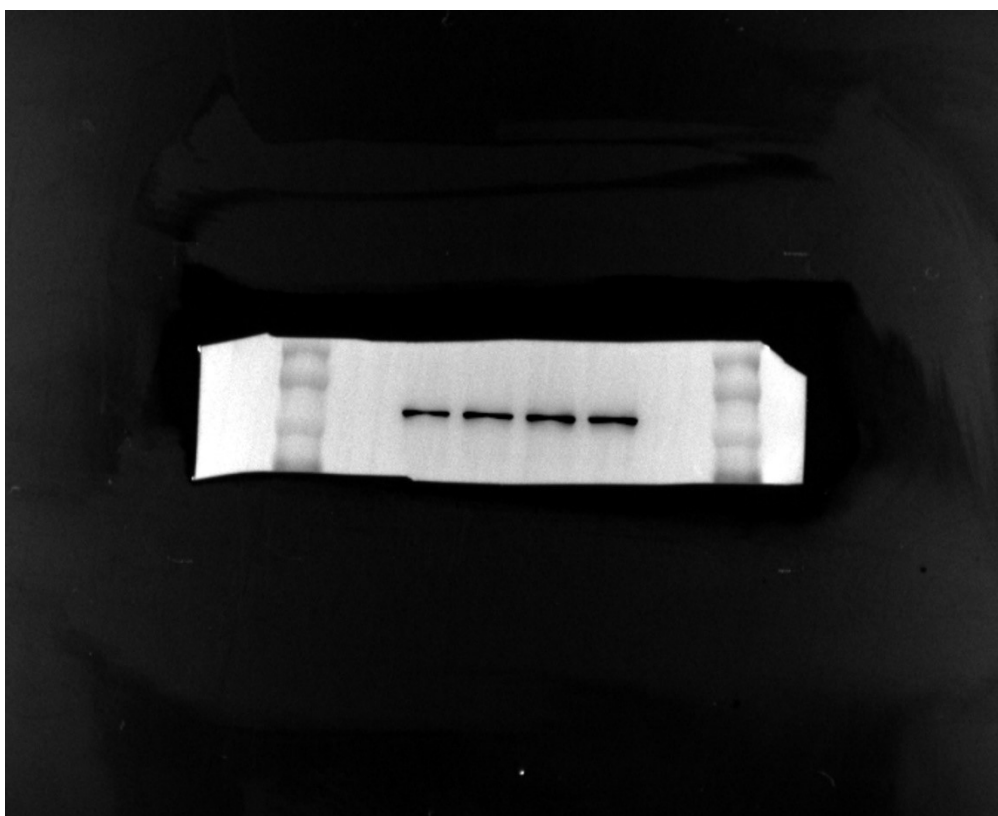

Huvecs CD62P

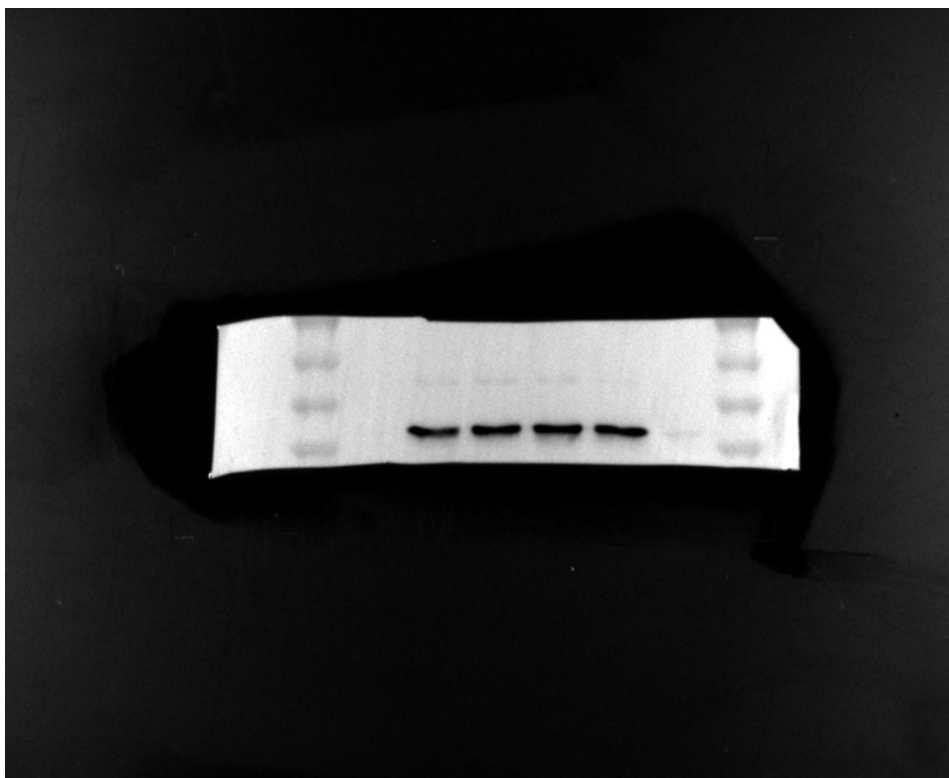

Huvecs GAPDH

Supplement: RA-015-D5RA02815A-s001 [file RA-015-D5RA02815A-s001.pdf]

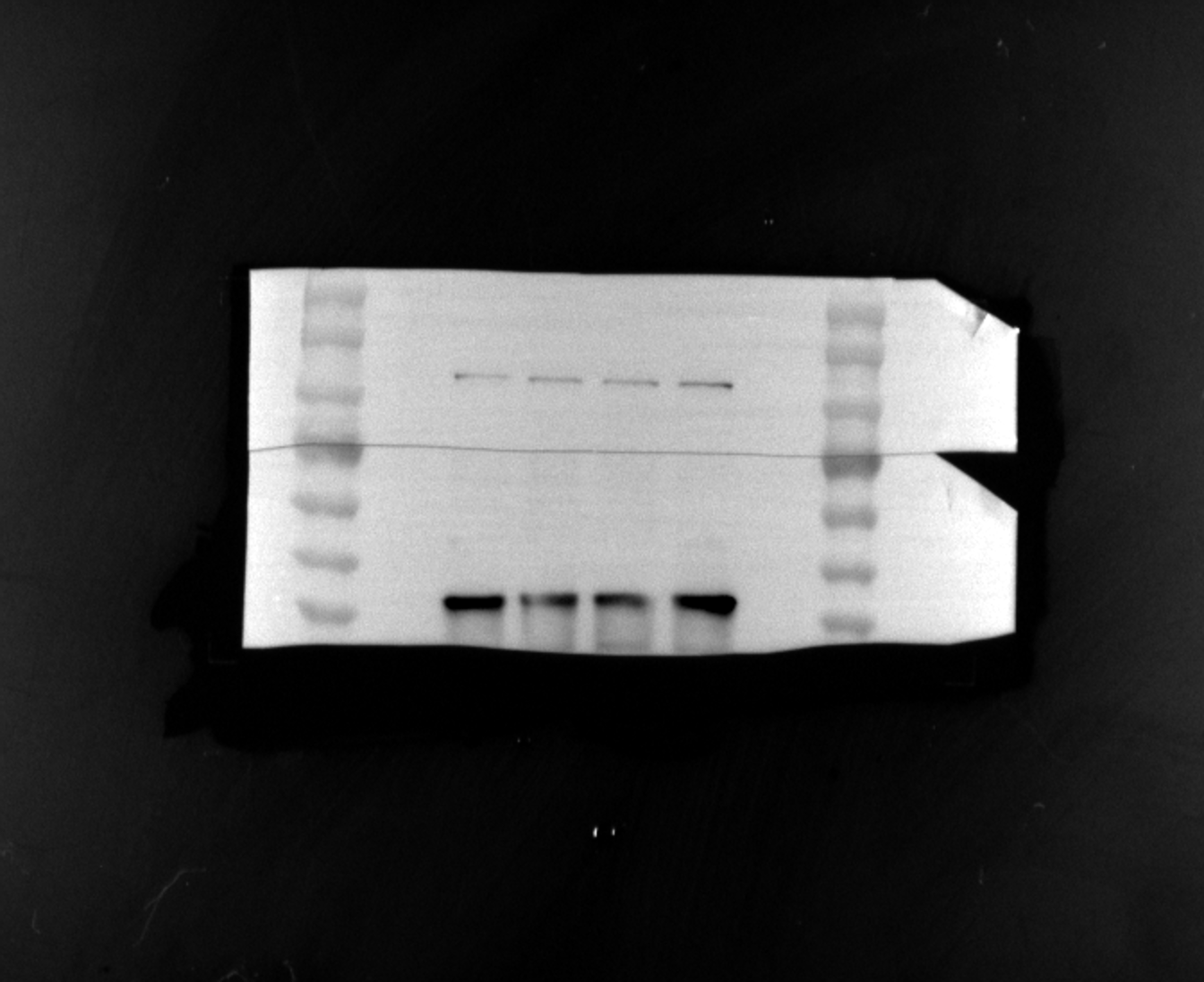

Supplement: RA-015-D5RA02815A-s002 [file RA-015-D5RA02815A-s002.zip › RSC western blots data/ct26/ct26 all.Tif]

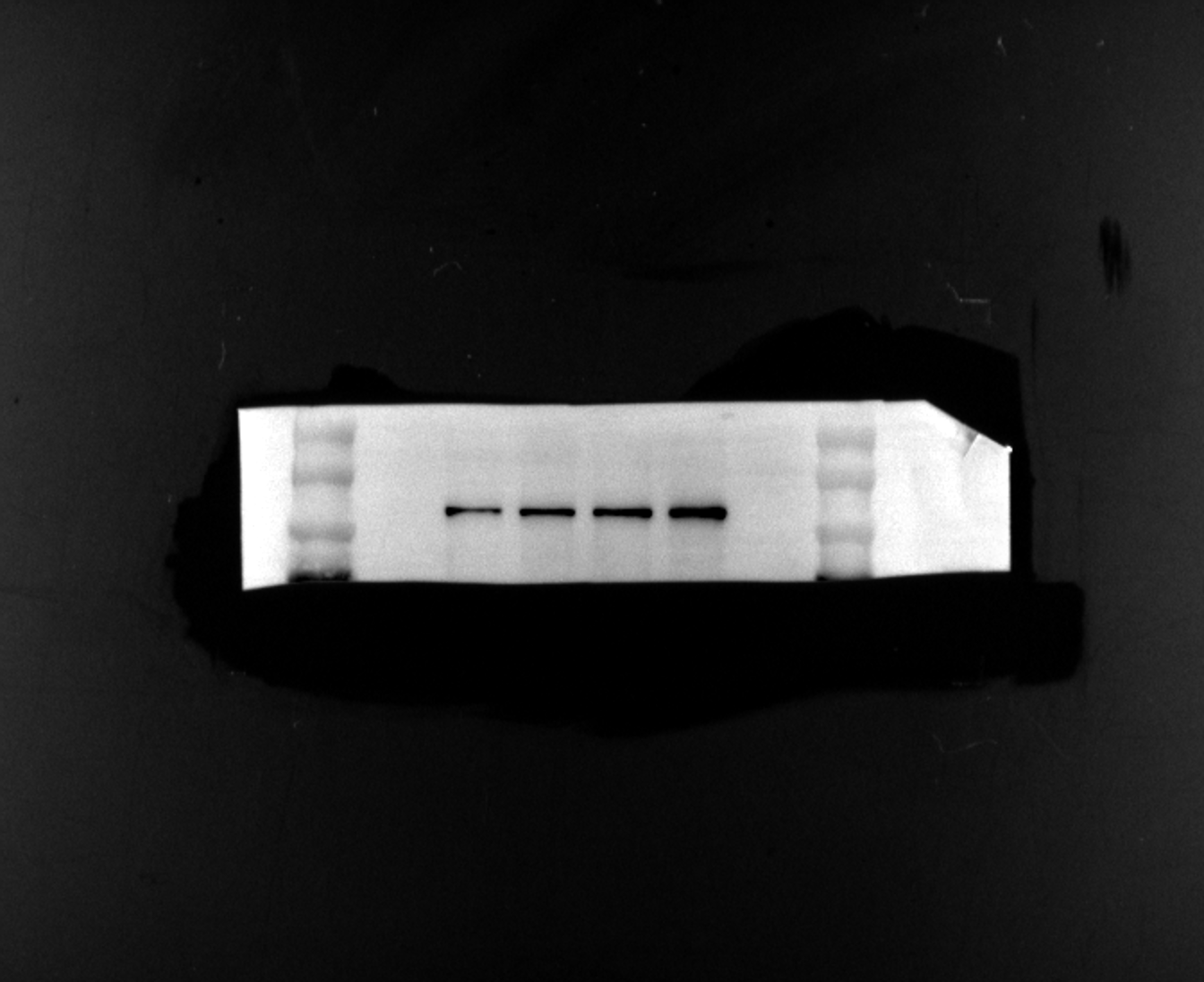

Supplement: RA-015-D5RA02815A-s002 [file RA-015-D5RA02815A-s002.zip › RSC western blots data/ct26/ct26 cd62p.Tif]

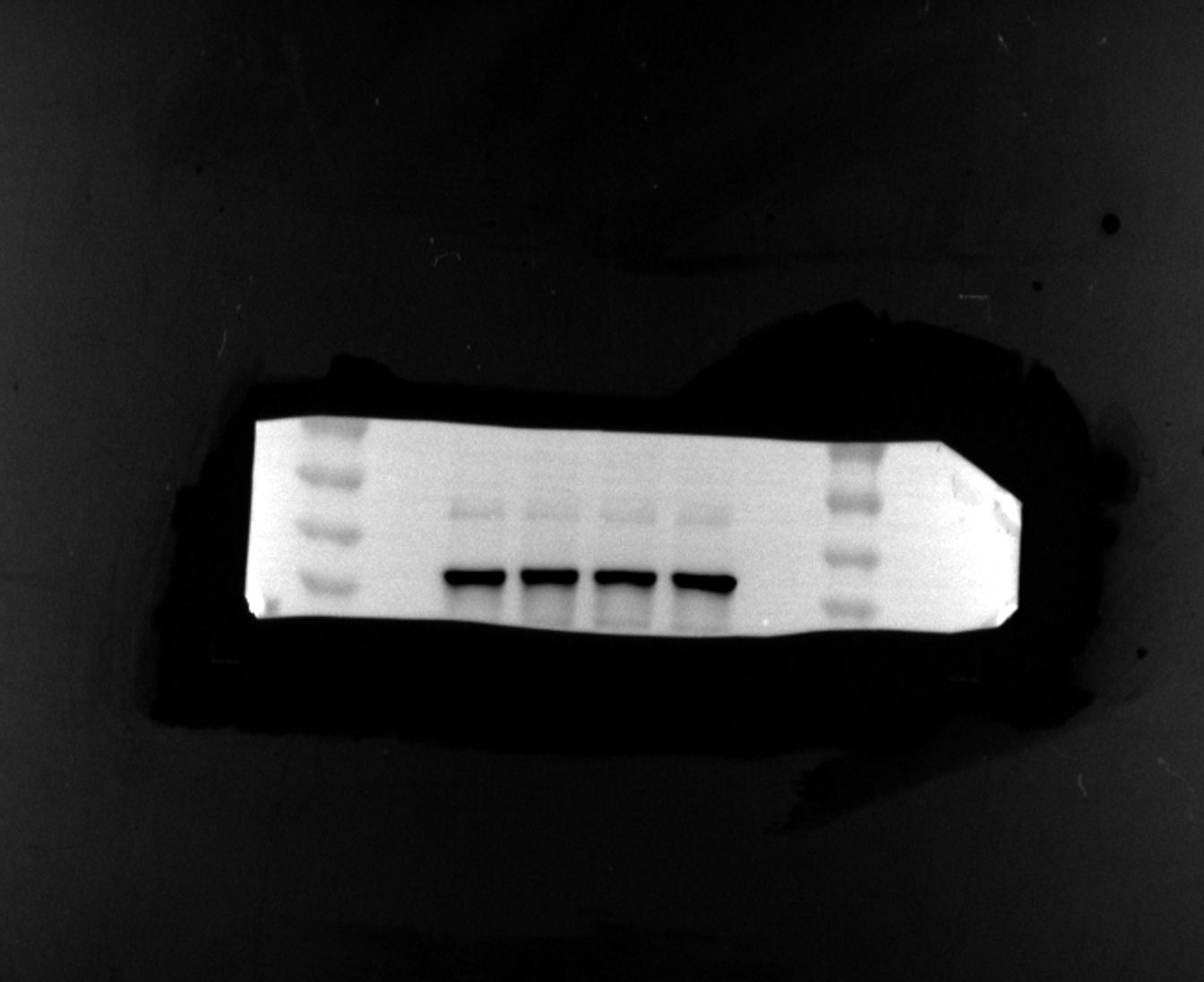

Supplement: RA-015-D5RA02815A-s002 [file RA-015-D5RA02815A-s002.zip › RSC western blots data/ct26/ct26 gapdh.Tif]

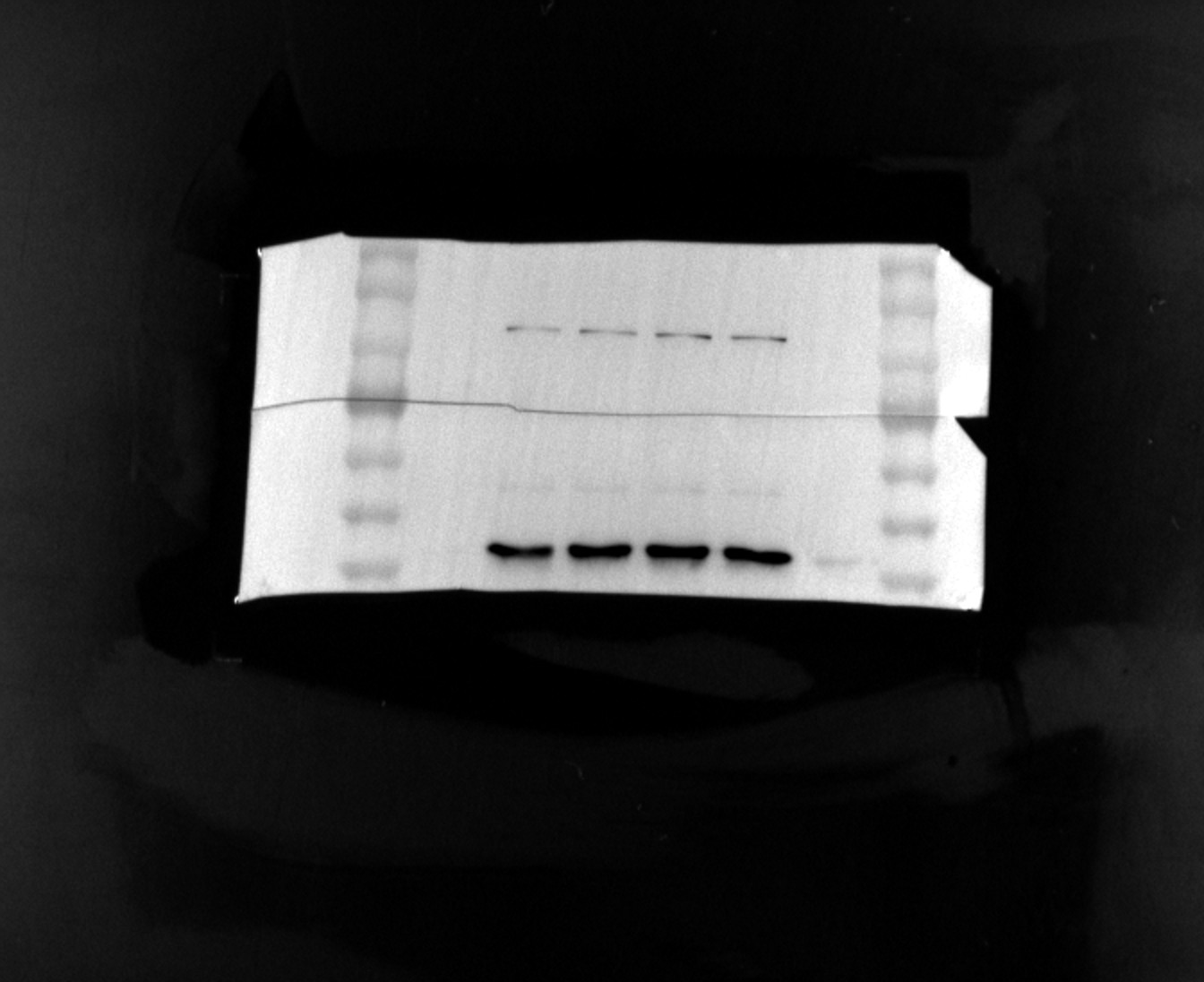

Supplement: RA-015-D5RA02815A-s002 [file RA-015-D5RA02815A-s002.zip › RSC western blots data/huvecs/all..Tif]

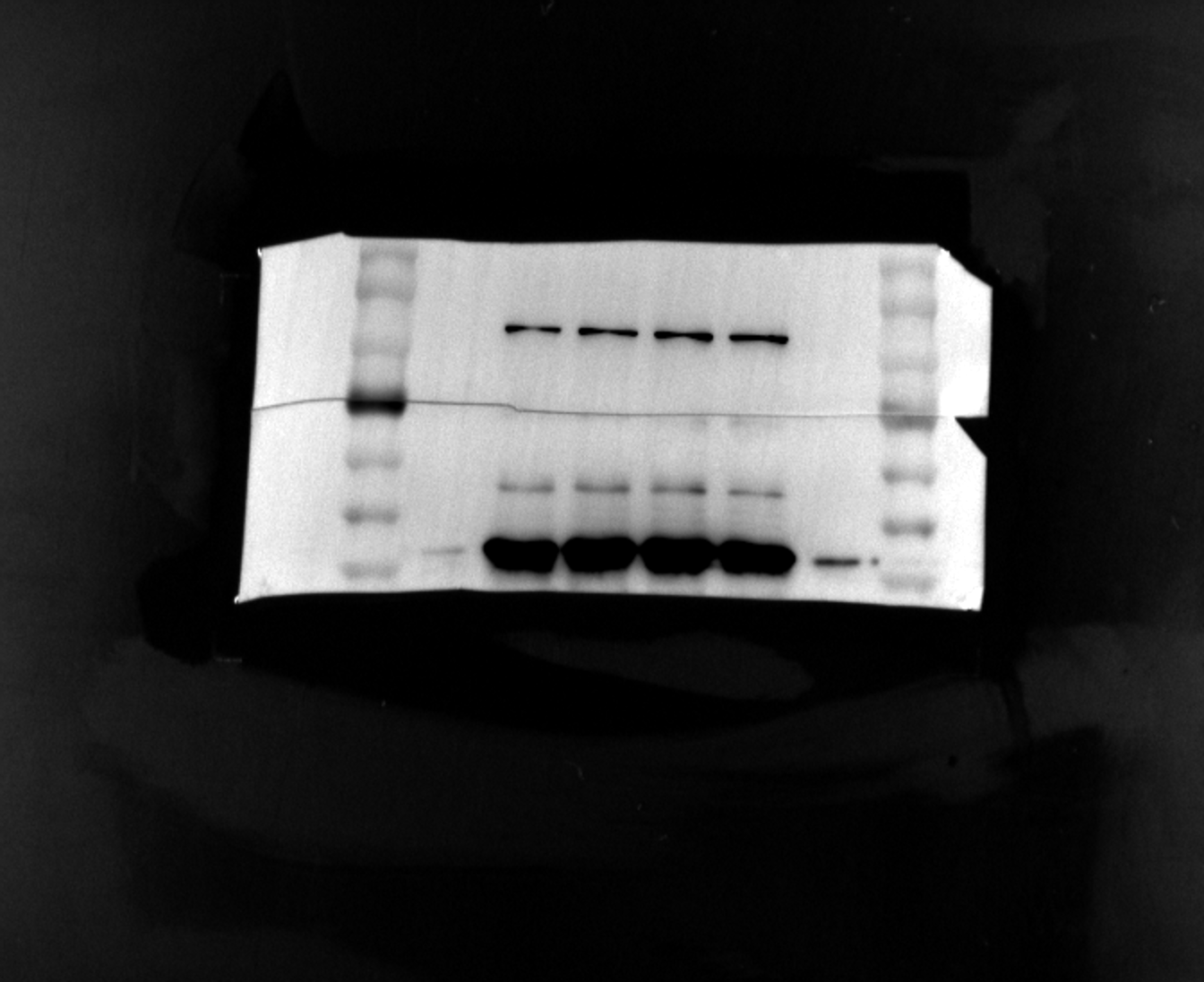

Supplement: RA-015-D5RA02815A-s002 [file RA-015-D5RA02815A-s002.zip › RSC western blots data/huvecs/all.Tif]

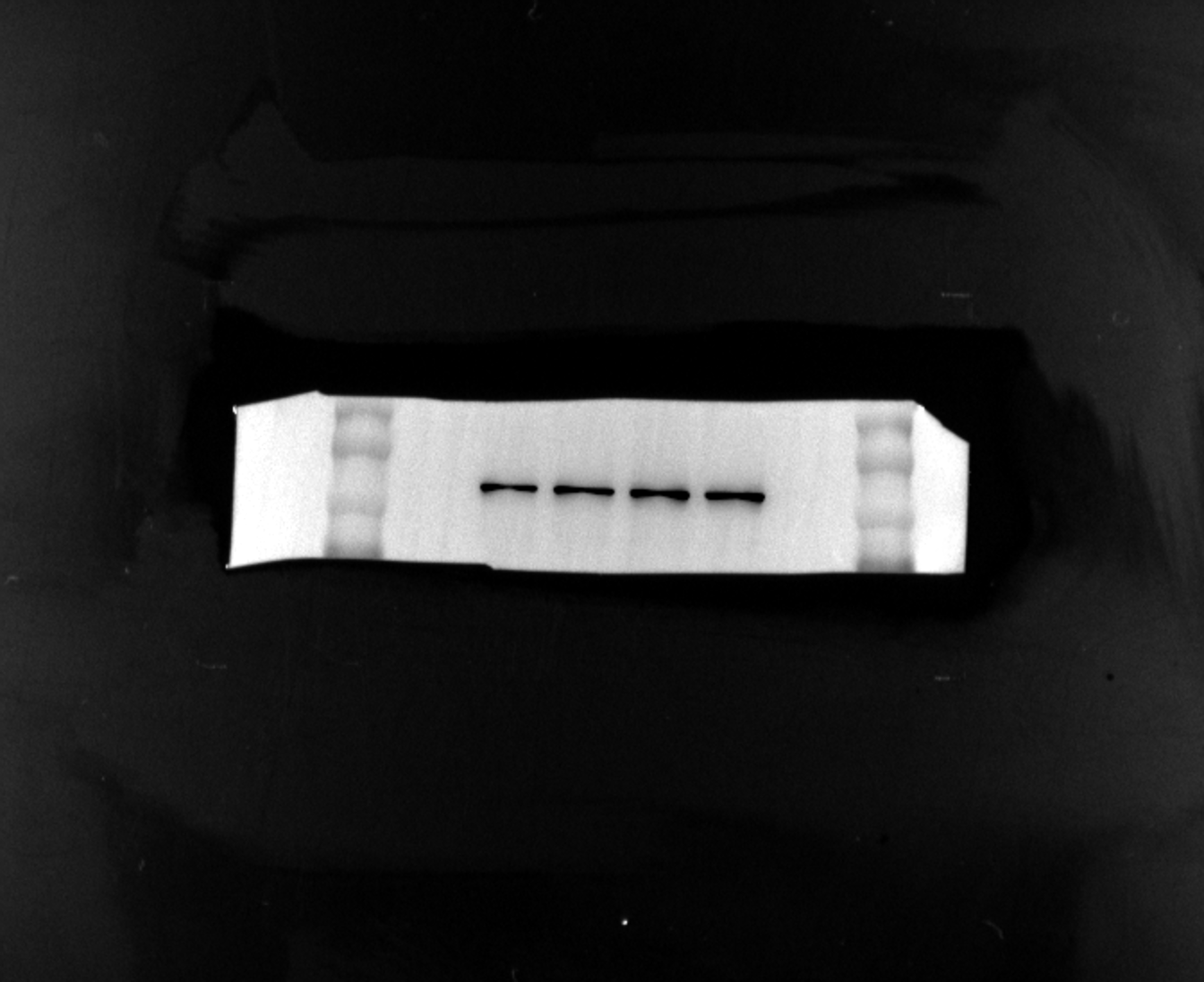

Supplement: RA-015-D5RA02815A-s002 [file RA-015-D5RA02815A-s002.zip › RSC western blots data/huvecs/cd62p.Tif]

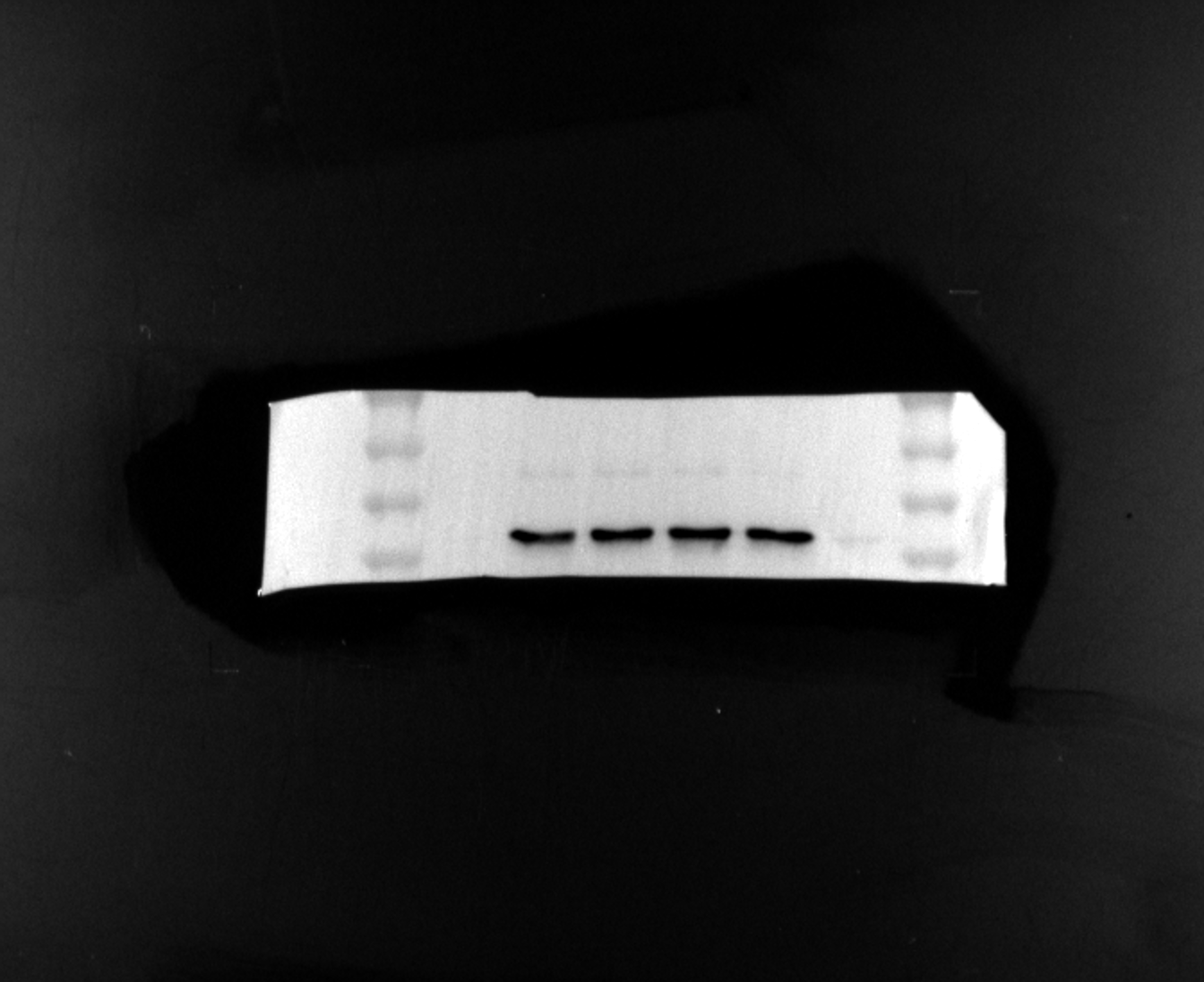

Supplement: RA-015-D5RA02815A-s002 [file RA-015-D5RA02815A-s002.zip › RSC western blots data/huvecs/gapdh.Tif]
